# Synthesis, Characterization, and Docking Studies of Some New Chalcone Derivatives to Alleviate Skin Damage Due to UV Light

**DOI:** 10.3390/molecules30051057

**Published:** 2025-02-25

**Authors:** Arun Kumar Mishra, Kamal Y. Thajudeen, Chandra Shekhar, Mhaveer Singh, Harpreet Singh, Arvind Kumar, Sarvesh Kumar Paliwal, Emdad Hossain, Shahana Salam

**Affiliations:** 1 SOS School of Pharmacy, Faculty of Pharmacy, IFTM University, Moradabad 244102, India; 2 Department of Pharmacognosy, College of Pharmacy, King Khalid University, Abha 611441, Saudi Arabia; 3 School of Pharmaceutical Sciences, Faculty of Pharmacy, IFTM University, Moradabad 244102, India; 4 Department of Pharmacy, Banasthali Vidyapith, Banasthali 304022, India; 5 Department of Pharmaceutical Technology, Jadavpur University, Kolkata 700032, India; 6 Department of Pharmaceutical Chemistry, Shri Jagdishprasad Jhabarmal Tibrewala University, Jhunjhunu 333053, India

**Keywords:** chalcone, aminochalcone, phenylacetamide, vanillin, 4-aminoacetophenone, sun protection factor (S.P.F.) activity, in vivo sunscreen activity

## Abstract

Increasing cases of sunburn is one of the serious problems across the globe. In this connection, there is an urgent requirement for some effective sun screening agents. In the search for the same, nanoemulsions of some new synthesized and characterized chalcone derivatives were prepared and evaluated in vitro and in vivo. In order to meet the said objective, in the first step, vanillin was reacted with 4-aminoacetophenone in the presence of 15% sodium hydroxide and ethanol to synthesize the target compounds (**C-1** to **C-5**). Progress of reaction was monitored using thin-layer chromatography (TLC). The crystals of purified compounds were characterized using spectroscopic techniques such as Infrared (IR) spectroscopy, ^1^H-NMR spectroscopy, ^13^C-NMR, and mass spectrometry. We prepared the nanoemulsions of the final compounds (**C-1** to **C-5**) and subsequently evaluated them for in vitro sun protection factor activity. The concentration of the nanoemulsions, consistently ranging from 0.88 to 0.91 mg/mL across all formulations, demonstrated a high degree of consistency. The range of particle size varied from approximately 172 to 183 nm, with low polydispersity index values (approximately 0.11 to 0.15). The negative zeta potentials recorded for all the formulations (ranging from −35.87 mV to −39.30 mV) showed that the nanoemulsions are electrostatically stable enough to keep them from sticking together. The pH values of the nanoemulsions ranged narrowly from approximately 5.00 to 5.16, which indicated the compatibility of emulsion with biological systems and the potential to reduce irritation or instability during administration. The viscosity of the nanoemulsions varied between 2.00 and 2.12 cP. In silico studies were performed using MMP-I and MMP-2 as target receptors. For in vitro SPF evaluation, the Mansur equation was employed. COLIPA guidelines were compiled for in vivo SPF evaluation. The nanoemulsions derived from compounds **C-3** and **C-4**, designated as **C-3NE** and **C-4NE**, were more effective as anti-aging agents. Findings suggested the possible scope of further synthesis of newer synthetic derivatives of chalcones for furfur development nanoemulsions for better SPF activity.

## 1. Introduction

In general, sunlight offers numerous advantages for human health; nevertheless, it can also produce detrimental effects. A number of studies have documented the detrimental effects of ultraviolet (UV) radiation from sunlight exposure. Workers in Tuscany found an increase in free radical concentration in their peripheral blood, an indicator of oxidative stress, during peak solar UV irradiance [1]. UV irradiation is both epidemiologically and molecularly associated with the three forms of skin cancer, which include basal cell carcinoma, squamous cell carcinoma, and malignant melanoma. Researchers noted mitochondrial impairment in human dermal cells after exposure to artificial sunlight as a source of UV light [2,3]. In vivo and in vitro studies showed that UV light in the boundary region (385–405 nm) causes much damage to skin cells and causes the formation of dark cyclobutene-pyrimidine dimers [4]. UV light can cause mutations that lead to melanoma and the activation of oncogenes [5]. Therefore, we must meticulously evaluate the safeguarding of human skin against direct UV irradiation exposure. Therefore, it is necessary to safeguard our skin surface from such harmful UV radiation.

In order to meet this requirement, some synthetic analogs of heterocyclic moiety must be explored. In the present investigation, chalcone moiety was selected to perform synthetic derivatization, and after docking and characterization, the formulation was prepared.

Chalcones have received significant attention because of their flavonoid nature and their attribute to inhibit free radicals [6]. Due to the presence of α,β-unsaturated ketone system, several biological activities are expressed by this class of compounds [7]. It is pertinent to mention that due to UV light exposure, ROS are generated, which in turn results in oxidative stress after skin damage. However, the phenomenon is reversed by chalcones and their derivatives. Other than this, the compounds with this moiety reduce the risk of mutations and skin cancer development [8]. Their ability to modulate DNA repair enzymes further enhances their photoprotective potential. Additionally, chalcones represent a promising natural moiety for skin protection against UV radiation due to their free radical scavenging activity, anti-inflammatory activity, and MMP-inhibitory characteristics [9,10,11]. With this basis, it is expected that their incorporation into sunscreens and formulations may result in an effective approach to mitigating photoaging. This provides a background and rationale for selecting the synthesis and evaluation of chalcone derivatives in the present context.

The chemical structure of chalcone typically consists of a *trans*-1,3-diphenyl-2-propen-1-one moiety, characterized as an α, β-unsaturated ketone [12]. Researchers refer to chalcones as antichlor pigments. Chalcones play a significant role in the pigmentation of the corolla in certain plants. They are also present in naturally occurring compounds, including plant allelochemicals, insect hormones, and pheromones [13]. They participate in various chemical reactions and are valuable in the synthesis of a range of heterocyclic compounds [14]. Chalcone serves as a prevalent scaffold in numerous biologically active compounds [15]. Chalcones demonstrated a range of biological and pharmacological activities, including antimicrobial, analgesic, anti-inflammatory, antimalarial, antiviral, and anticancer effects [16,17,18,19,20,21]. Chalcones represent a class of compounds characterized by significant antioxidant activity, especially concerning skin health [22].

Keeping in view the possible pharmacological attributes of chalcone derivatives, we worked to synthesize some new chalcone derivatives and prepared O/W nanoemulsion after due characterization and docking. For the synthesis of new chalcone derivatives, condensation of aryl ketone with aromatic Aldehyde was performed. Further, nanoemulsions were evaluated for their sun protection activity against UV light using in vivo and in vitro models.

## 2. Results

### 2.1. Synthesis

**Scheme 1:**
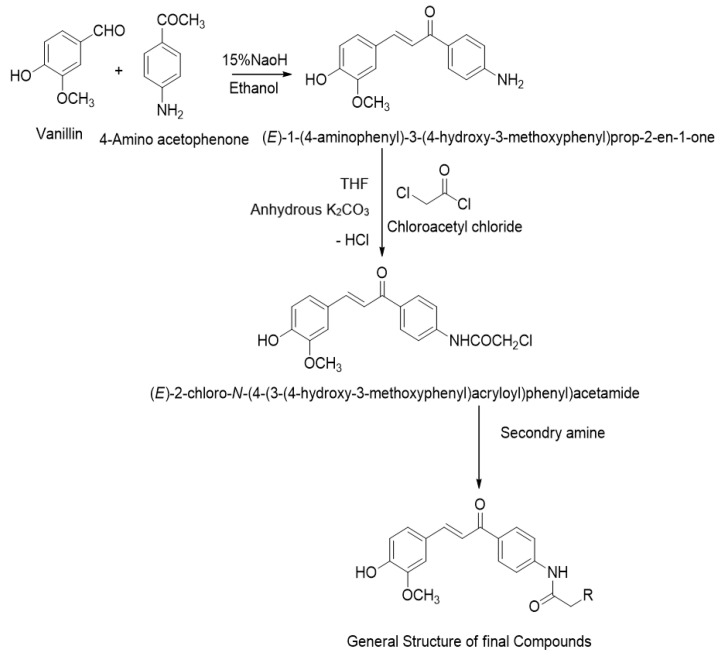
Synthesis of chalcone derivatives. R = Piperazine (**C-1**), Methyl Piperazine (**C-2**), Ethylpiperazine (**C-3**), Diphenylamine (**C-4**), Pyrroliddine (**C-5**).

The new chalcone derivatives were successfully synthesized using Scheme 1. A 15% sodium hydroxide solution facilitated the reaction between vanillin and 4-amino acetophenone in ethanol, yielding pure (*E*)-1-(4-aminophenyl)-3-(4-hydroxy-3-methoxyphenyl) prop-2-en-1-one (**I-1**). Using anhydrous potassium carbonate, the intermediate I was reacted with chloroacetyl chloride in tetrahydrofuran to yield (*E*)-2-Chloro-*N*-(4-(3-(4-Hydroxy-Methoxyphenyl) acryloyl) phenyl) acetamide (**I-2**). The intermediate **I-2** was mixed with several secondary amines in acetone with the help of anhydrous potassium carbonate to make the final products **C-1**, **C-2**, **C-3**, **C-4**, and **C-5**. We obtained the synthesized derivatives in solid form, with yields ranging from 58% to 80%. We confirmed the purity and uniformity of all synthesized derivatives through their melting point and thin-layer chromatography (TLC). Table 1 displays the physical characterization data of the synthesized compounds. The recrystallization of synthesized derivatives was performed using ethanol as a solvent. The structure of all synthesized compounds was confirmed using IR, ^1^HNMR, ^13^CNMR, and mass spectrometry.

### 2.2. Docking

We simulated the ligand compounds (**C-1** to **C-5**) for docking at Matrix Metalloproteinase-1 (MMP-1) (PDB: 3shi), Matrix Metalloproteinase-2 (MMP-2) (PDB: 8h78). In the present investigation, vanillin and oxybenzone were employed as control agents. The rationale behind the usage of vanillin and oxybenzone as control agents lies in the fact that vanillin and its derivatives have the potential to absorb ultraviolet radiation, especially in the UV-B range. Other than this, it is also important to record that vanillin reduces the inflammation induced by UV light exposure and ultimately reduces erythema and edema, including skin irritation. Oxybenzone potentiates the conversion of harmful UV-B light to less harmful heat energy, and this, in turn, causes a lower risk of sunburn and results in skin protection. Based upon the literature review, it was elucidated that oxybenzone is combined with another sunscreen agent to enhance the SPF of formulations several folds.

The reason for selecting the MMP-1 and MMP-2 for in silico studies on UV-induced skin damage is because of their critical roles in extracellular matrix (ECM) degradation and skin aging. The literature suggests that MMP-1 causes degradation of collagen type I and III as these are required as essential components for skin elasticity. On the other hand, activation of MMP-2 gets stimulated due to UV-induced oxidative stress. As the inhibition of MMP-1 and MMP-2 results in the prevention of collagen breaking, therefore the in silico study will provide insights into mitigating UV-induced skin damage and assist in developing skincare treatments.

**Figure 1:**
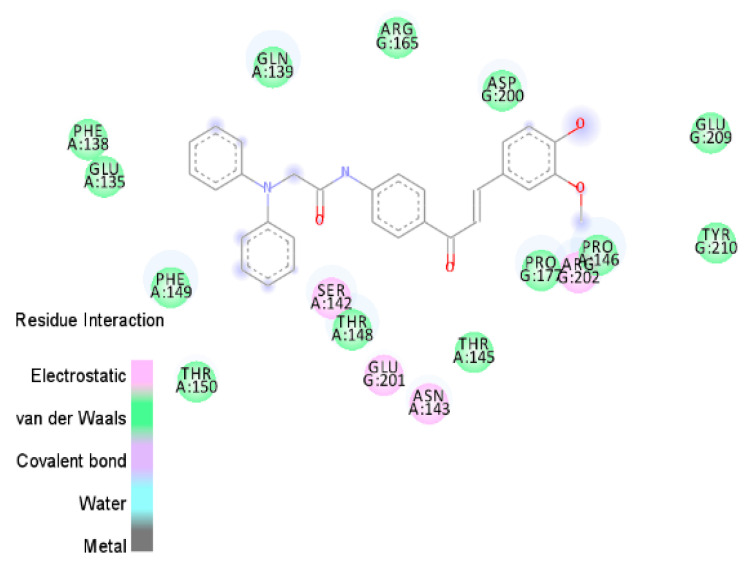
Two-dimensional view of molecular interaction of C4 with PDB: 8H78 of MMP1.

**Figure 2:**
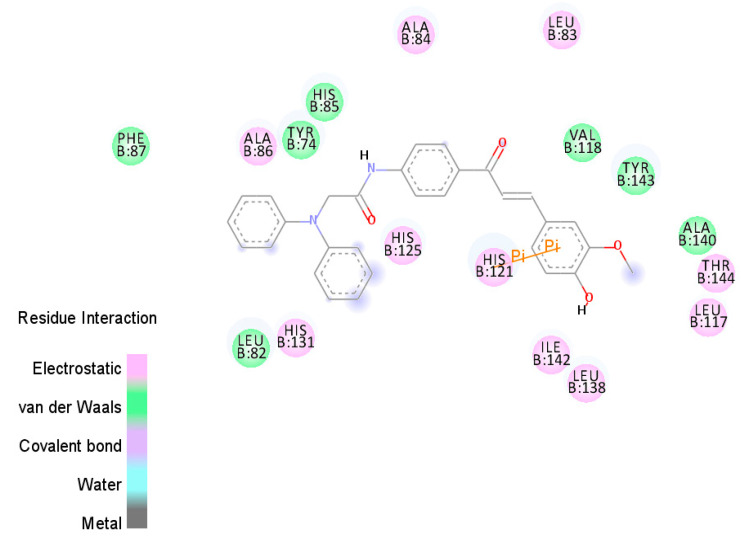
Two-dimensional view of molecular interaction of C4 with PDB: 8H78 of MMP2.

We conducted a total of 14 blind docking studies, which enabled ligands to independently explore their binding sites and poses without any bias toward the binding pocket [23,24]. Each docking simulation yielded nine poses. We selected the optimal docked conformation to illustrate molecular interactions in both 2D and 3D representations. Docked compounds were located in the hydrophobic region of their binding site. The synthesized molecules exhibited superior binding affinity compared to the control molecules (Table 2). In MMP-1, positive controls exhibited interactions with HIS-218 (via pi-interaction) and LEU-181 (through hydrogen bond interaction). **C-2** exhibited pi-interaction with PRO-146. C5 exhibited numerous PI interactions with ARG and PRO. Consequently, it can be inferred that **C-1**–**C-5** exhibits stronger binding than vanillin and oxybenzone, albeit through distinct mechanisms of action. Vanillin had a pi-bond interaction with HIS-121 for MMP-2, while oxybenzone had a pi-bond interaction with HIS-121 and hydrogen bond interactions with ILE-142 and ALA-140. Similar to vanillin, the compounds **C-1**, **C-2**, **C-3**, **C-4**, and **C-5** also demonstrated pi-bond interactions with HIS-121. Therefore, it was concluded that **C-1**–**C-5** exhibits the same mechanism of action as vanillin when binding to MMP-2 (Table 3). Table 2 and Table 3 display the binding energies of the synthesized derivatives. Figure 1 and Figure 2 represent the dock poses of C4 with MMP-1 and MMP-2, respectively. Figures S1–S14 display the docking images of synthesized compounds with MMP-1 and MMP-2.

The nanoemulsions were prepared as per the formula presented in Table 3. To prepare the nanoemulsion, both phases (oil and water) were combined together at high temperatures (80–85 °C) and with vigorous agitation in the presence of an emulsifier until the oil droplets were completely dispersed within the water as a dispersion phase.

### 2.3. Findings of Physical Parameters of Nanoemulsions

The physical characteristics of nanoemulsions derived from **C-1**, **C-2**, **C-3**, **C-4**, and **C-5** revealed a meticulous design for both functional and sensory attributes. The cream color and agreeable fragrance enhanced the product’s aesthetic allure and user contentment, rendering it a comprehensive and interesting formulation for diverse cosmetic and pharmaceutical uses. Table S1 (Supplementary Materials) displays the physical properties of nanoemulsions and pictorial presentation.

### 2.4. Identification of Nanoemulsions

We conducted the dilution test, dye solubility test, and cobalt chloride test to identify the types of prepared nanoemulsion formulations. All tests indicated that the prepared nanoemulsion formulations were of the oil-in-water (o/w) type.

### 2.5. Physicochemical Properties of Nanoemulsions

Nanoemulsions **C-1NE**, **C-2NE**, **C-3NE**, **C-4NE**, **C-5NE**, and the blank formulation have physicochemical properties that helped us understand their properties, which are important for many uses.

### 2.6. Particle Size (nm)

The particle size of nanoemulsions is a crucial parameter that affects their stability and efficacy in the delivery of active ingredients. Nanoemulsions **C-1NE**, **C-3NE**, **C-4NE**, and **C-5NE** demonstrated particle sizes ranging from approximately 172.30 to 183.0 nanometers, with **C-1NE** identified as the smallest at 172.30 nm. **C-2NE** exhibited a marginally larger particle size of 183.00 nm. This range places the blank formulation at 172.57 nm. Nanoscale dimensions are advantageous due to their ability to improve solubility and bioavailability, rendering them appropriate for a range of applications.

### 2.7. Polydispersity Index

The polydispersity index (PDI) quantifies the uniformity of particle size distribution in a formulation. Lower PDI values, as observed in this study (ranging from 0.11 to 0.15), suggested a relatively narrow and uniform distribution. The observed uniformity indicates that these nanoemulsions possess a well-structured nature, reducing the likelihood of particle aggregation or separation.

### 2.8. Zeta Potential (mV)

Zeta potential (mV) indicates the electrostatic repulsion among particles in a dispersion, which affects the stability of nanoemulsions. The values for **C-1NE**, **C-2NE**, **C-3NE**, **C-4NE**, and **C-5NE** were between −37.90 and −39.30 mV, which suggested strong electrostatic repulsion that prevented the particles from sticking together. The blank formulation demonstrated a zeta potential of −35.87 mV, indicating a slightly lower value while remaining within a stable range.

### 2.9. pH

The pH level of nanoemulsions is crucial for their compatibility with diverse applications, such as skincare and pharmaceuticals. The pH values of all nanoemulsions analyzed were near neutral, ranging from 5.09 to 5.16, while the blank exhibited a pH of 5.00. The near-neutral pH range is typically effective in reducing skin irritation and ensuring compatibility with various products.

### 2.10. Viscosity (cP)

Viscosity quantifies the thickness or flow resistance of a liquid, influencing the ease of application. The viscosity values for these nanoemulsions were relatively low, ranging from 2.00 cP to 2.12 cP. This indicates that these formulations possess moderate viscosity, facilitating ease of application and spreading. Table S2 (Supplementary Materials) details the physicochemical characteristics of nanoemulsions.

The physicochemical properties of nanoemulsions **C-1NE**, **C-2NE**, **C-3NE**, **C-4NE**, **C-5NE**, and the blank showed that they are stable, well structured, and useful for many things. To safely deliver active ingredients, nanoemulsions with small particles, low polydispersity indices, strong zeta potentials, pH values close to neutral, and moderate viscosities are good choices. The blank formulation provided a useful benchmark value for the comparison of these characteristics.

### 2.11. In Vitro SPF Value of Nanoemulsions

We assessed the SPF values for five distinct nanoemulsion formulations—**C-1NE**, **C-2NE**, **C-3NE**, **C-4NE**, and **C-5NE**—and found a range from 2.308 to 8.798. Table 4 displays the results. Average absorbance values of triplicate experiments were subjected to multiplication with EE and I on different wavelengths, which, in turn, resulted in the summation value as SPF.

### 2.12. In Vivo Skin Irritation Activity

#### 2.12.1. Erythema Reaction

The erythema testing results for the sunscreen nanoemulsions (**C-1NE**, **C-2NE**, **C-3NE**, **C-4NE**, and **C-5NE**), the standard Oxibenzone sunscreen cream, and the negative control resulted in significant insights into the potential for skin irritation and the safety profiles of these products. The findings of in vivo skin irritation activity demonstrated that the Primary Irritation Index (PII) for all formulations varies from 0.55 to 0.90, indicating minimal irritation, as outlined in Table S5 (Supplementary Materials).

#### 2.12.2. In Vivo SPF Evaluation

Related to the in vivo SPF study, it was found that **C-4NE** and **C-3NE** exhibited the highest SPF values of 7.20 and 5.39, respectively; however, **C-1NE** showed the lowest SPF value of 2.59. The results are presented in Table S4 (Supplementary Materials).

## 3. Discussion

**C-1NE** and **C-2NE** demonstrated SPF values of 2.582 and 2.539, respectively. These formulations offer a limited degree of UV protection, categorizing them within the lower range of the SPF spectrum. These products may be appropriate for daily use or circumstances involving minimal sun exposure; however, they may not provide sufficient protection during prolonged periods of intense sunlight.

**C-5NE**, exhibiting an SPF value of 2.308, aligns with the SPF range of **C-1NE** and **C-2NE**. The product offers a moderate level of protection appropriate for daily use; however, it may not be the best option for activities that necessitate higher sun protection levels.

**C-3NE** exhibited a notably higher SPF value of 6.876, demonstrating its enhanced UV protection efficacy. **C-3NE** appears to be a viable nanoemulsion formulation for those requiring moderate sun protection. This formulation may be advantageous in everyday skincare products, providing a balance of protection while maintaining a lightweight texture on the skin.

**C-4NE** was identified as the most effective nanoemulsion, exhibiting a notable SPF value of 8.798. This formulation provides effective protection against UVB radiation, making it suitable for prolonged sun exposure or for those relatively more prone to sunburn but with low sunlight intensity.

Finally, the in vitro SPF values distinctly differentiated the various nanoemulsion formulations. **C-4NE**, possessing the highest SPF value, is the optimal selection for scenarios necessitating maximum sun protection. **C-3NE** is acknowledged as an effective nanoemulsion formulation with significant UV protection properties. The selection of nanoemulsion must take into account SPF values, skin compatibility, the required level of protection, and specific application needs to achieve effective sun protection and user satisfaction.

## 4. Materials and Methods

All chemicals utilized in the present investigation were procured from commercial sources such as Central Drug House, New Delhi, and S.D. Fine, Mumbai, India, employed without additional purification. Recrystallization was performed utilizing ethanol as the solvent. TLC was employed to assess the purity of newly synthesized derivatives. Ethyl acetate and hexane were utilized as a solvent system in a 1:1 ratio. The open capillary tube method was employed to determine the melting point of intermediates and newly synthesized compounds. Infrared spectra were obtained using an FTIR spectrometer (Bruker Corporation, Billerica, MA, USA) employing either KBr pellets or the neat product. ^1^H and ^13^C NMR spectra were recorded using a Bruker AV 400 MHz spectrometer (Bruker Corporation, Billerica, MA, USA). Chemical shifts (δ) are expressed in parts per million (ppm), while coupling constants (J) are indicated in Hertz (Hz). The splitting patterns of NMR signals are classified as follows: br for broad, s for singlet, d for doublet, t for triplet, q for quartet, and m for multiplet. The mass spectra were recorded on LC-MSD-Trap-SL (Agilent Technologies, Bengaluru, India). The Malvern particle size analyzer was utilized to determine particle size and zeta potential at a temperature of 25 °C.

### 4.1. Synthesis of Compounds

#### 4.1.1. Synthesis of (*E*)-1-(4-Aminophenyl)-3-(4-hydroxy-3-methoxyphenyl)prop-2-en-1-one (**I-1**)

Vanillin (0.01 M) and 4-amino acetophenone (0.01 M) were dissolved in 100 mL of ethanol with the addition of 15% sodium hydroxide in a beaker. The solution was maintained in an ice bath and stirred with magnetic beads for 12 h at a temperature of 0–5 °C. The mixture was subjected to stirring for 12 h, followed by filtration and the addition of ice-cold water. The resulting solid was washed with cold distilled water, dried, and recrystallized using ethanol. % Yield = 59.27, M.P. = 65–67 °C, Rf. Value: 0.50; ^1^H-NMR (500 MHz, CDCl_3_) (ppm): 3.84 (s, 3H, OCH_3_), 4.68 (s, 2H, NH_2_), 6.55–7.12 (m, 7H, Ar), 8.12 (s, 1H, OH); ^13^C-NMR (125 MHz, CDCl_3_) (ppm): 55.1, 113.65, 122.8, 127.39, 130.82, 151.64, 196.79, IR (KBr) cm^−1^: 1123 (C-C Str.), 1178 (C-O Str.), 1243 (O-H Str.), 1354 (C=C Str.), 1616 (C-N Str.), 1695 (C=O Str.), 3420 (C-H Str.), 3428 (N-H Str.); MS (FAB) [M + 1]+: (*m*/*z*): 270.12.

#### 4.1.2. Synthesis of (*E*)-2-Chloro-*N*-(4-(3-(4-hydroxy-3-methoxyphenyl) acryloyl)phenyl) acetamide (**I-2**)

In the presence of anhydrous K_2_CO_3_, the equivalent mole of compound-1 was dissolved in THF (Tetrahydrofuran). After this, the reaction mixture was refluxed at 40–45 °C for 15 h. The chloroacetyl chloride mixture was subsequently introduced in drops. To obtain the precipitate, the reaction mixture was concentrated, filtered, washed with cold water, dried, and recrystallized from ethanol. % Yield = 66.39, M.P. = 110–112 °C, R_f_. Value: 0.36; ^1^H-NMR (500 MHz, CDCl_3_) (ppm): 3.86 (s, 3H, OCH_3_), 4.21 (s, 2H, NH_2_), 6.54 (s, 1H, CONH), 7.12–9.61 (m, 7H, Ar), 8.53 (s, 1H, OH); ^13^C-NMR (125 MHz, CDCl_3_) (ppm): 42.92, 164.24, 197.02, 77.06, 113.97, 119.34, 129.75, 130.84, 133.68, 141.06; IR (KBr) cm^−1^: 816 (C-Cl Str.), 1133 (C-C Str.), 1178 (C-O Str.), 1236 (O-H Str.), 1349 (C=C Str.), 1634 (C=O Str.), 3150 (C-H Str.), 3501 (N-H Str.), 3501 (C-N Str.); MS (FAB) [M + 1]+: (*m*/*z*): 346.11.

#### 4.1.3. Synthesis of Chalcone Derivatives (**C-1** to **C-5**)

Compound-2 (0.01M) and substituted secondary Amine (0.01M) were dissolved in 100 mL of acetone in a 250 mL RBF apparatus. The reaction was completed by refluxing the reaction mixture for 14–16 h. TLC was employed to monitor the reaction’s progression. Next, the reaction mixture was cooled and allowed to stand overnight to facilitate precipitation. The precipitated compound was filtered, washed with water, and recrystallized from ethanol before being dried. Table 1 contains the physical characterization data of the synthesized compounds.

#### 4.1.4. (*E*)-*N*-(4-(3-(4-Hydroxy-3-methoxyphenyl) acryloyl)phenyl)-2-(piperazin-1-yl)acetamide (**C-1**)

^1^H-NMR (500 MHz, CDCl3) (ppm): 1.62–2.10 (s, 5H, Piperazine), 3.43 (s,2H, CH_2_), 3.89 (s, 3H, OCH_3_), 6.50 (s, 1H, CONH), 7.85 (d, *J* = 4, 2H, Ar), 7.87 (d, *J* = 4.25, 2H, Ar), 7.15 (s, 1, Ar), 6.99 (d, *J* = 4.75, 1H, Ar), 6.78 (d, *J* = 5.25, 1H, Ar), 9.40 (s, 1H, OH); ^13^C-NMR (125 MHz, CDCl_3_) (ppm): 45.82, 54.12, 62.53, 77.07, 113.70, 118.72, 118.79, 127.76, 129.79, 130.81, 132.93, 141.80, 168.69, 196.93; IR (KBr) cm^−1^: 1121 (C-O Str.), 1260 (O-H Str.), 1362 (C=C Str.), 1622 (C-N Str.), 1635 (C=O Str.), 1652 (C-C Str.), 3019 (C-H Str.); MS (FAB) [M + 1]+: (*m*/*z*): 396.10.

#### 4.1.5. (*E*)-*N*-(4-(3-(4-Hydroxy-3-methoxyphenyl)acryloyl)phenyl)-2-(4-methylpiperazin-1-yl) acetamide (**C-2**)

^1^H-NMR (500 MHz, CDCl_3_) (ppm): 2.26 (s, 5H, CH_3_), 2.65 (s, 4H, Piperazine), 3.48 (s, 2H, CH_2_), 3.98 (s, 3H, OCH_3_), 6.62 (s, 1H, CONH), 7.88 (d, *J* = 3.75, 2H, Ar), 7.75 (d, *J* = 4.50, 2H, Ar), 7.24 (s, 1, Ar), 6.81 (d, *J* = 4.50, 1H, Ar), 6.89 (d, *J* = 4.75, 1H, Ar), 9.56 (s, 1H, OH); ^13^C-NMR (125 MHz, CDCl_3_) (ppm): 26.44, 45.15, 52.33, 54.65, 61.60, 113.70, 118.81, 127.72, 129.75, 130.81, 132.93, 141.87, 151.31, 168.42, 196.58; IR (KBr) cm^−1^: 1121 (C-O Str.), 1266 (O-H Str.), 1464 (C=C Str.), 1489 (C-C Str.), 1539 (C-N Str.), 1622 (C=O Str.), 3100 (C-H Str.); MS (FAB) [M + 1]+: (*m*/*z*): 410.09.

#### 4.1.6. (*E*)-2-(4-Ethyl piperazine-1-yl)-*N*-(4-(3-(4-hydroxy-3-methoxyphenyl)acryloyl)phenyl) acetamide (**C-3**)

^1^H-NMR (500 MHz, CDCl_3_) (ppm): 1.10–2.23 (s, 5H, C_2_H_5_), 2.30–2.35 (s, 4H, Piperazine), 3.38 (s, 2H, CH_2_), 3.89 (s,3H, OCH_3_), 6.43 (s, 1H, CONH), 7.92 (d, *J* = 5.75, 2H, Ar), 7.80 (d, *J* = 3.75, 2H, Ar), 7.73 (s, 1, Ar), 6.85 (d, *J* = 1.25, 1H, Ar), 6.70 (d, *J* = 5, 1H, Ar),9.62 (s, 1H, OH); ^13^C-NMR (125 MHz, CDCl_3_) (ppm): 10.57, 51.59, 51.98, 52.14, 61.44, 113.67, 118.97, 127.51, 129.67, 130.80, 132.85, 142.05, 151.52, 168.41, 196.63; IR (KBr) cm^−1^: 1121 (C-O Str.), 1260 (O-H Str.), 1456 (C=C Str.), 1472 (C-C Str.), 1539 (C-N Str.), 1616 (C=O Str.), 3019 (C-H Str.); MS (FAB) [M + 1]+: (*m*/*z*): 424.13.

#### 4.1.7. (*E*)-2-(Diphenylamine)-*N*-(4-(3-(4-hydroxy-3 methoxyphenyl)acryloyl)phenyl)acetamide (**C-4**)

^1^H-NMR (500 MHz, CDCl_3_) (ppm): 3.63 (s,3H, CH_3_), 4.11 (s,2H, CH_2_) 6.88 (s, 1H, CONH), 7.87 (d, *J* = 5.0, 2H, Ar), 7.79 (d, *J* = 2.0, 2H, Ar), 7.74 (s, 1, Ar), 7.31 (d, *J* = 2. 50, 4H, Ar), 7.20 (d, *J* = 7. 25, 4H, Ar), 7.09 (d, *J* = 5, 2H, Ar),6.81 (d, *J* = 2. 50, 1H, Ar), 6.73 (d, *J* = 5, 1H, Ar),8.62 (s, 1H, OH); ^13^C-NMR (125 MHz, CDCl_3_) (ppm): 76.79, 77.05, 117.87, 119.31, 121.07, 129.34, 130.78, 133.70, 140.99, 143.06, 164.16, 196.96; IR (KBr) cm^−1^: 1120 (C-O Str.), 1244 (O-H Str.), 1336 (C=C Str.), 1472 (C-C Str.), 1616 (C-N Str.), 1116 (C=O Str.), 2919 (C-H Str.), 3420 (N-H Str.); MS (FAB) [M + 1]+: (*m*/*z*): 479.01.

#### 4.1.8. (*E*)-*N*-(4-(3-(4-Hydroxy-3-methoxyphenyl)acryloyl)phenyl)-2-(pyrrolidin-1-yl)acetamide (**C-5**)

^1^H-NMR (500 MHz, CDCl_3_) (ppm): 3.61 (s, 3H, CH_3_), 4.09 (s, 2H, CH_2_) 6.79 (s, 1H, CONH), 7.88 (d, *J* = 4.0, 2H, Ar), 7.76 (d, *J* = 5.25, 2H, Ar), 7.69 (s, 1, Ar), 6.89 (d, *J* = 2.0, 1H, Ar), 6.73 (d, *J* = 3.75, 1H, Ar), 8.58 (s, 1H, OH); ^13^C-NMR (125 MHz, CDCl_3_) (ppm): 21.87, 24.0, 54.52, 56.32, 59.38, 76.98, 113.52, 120.17, 122.41, 127.71, 129.63, 132.66, 142.21, 151.49, 157.63, 169.03, 196.51; IR (KBr) cm^−1^: 1120 (C-O Str.), 1244 (O-H Str.), 1308 (C=C Str.), 1472 (C-C Str.), 1589 (C-N Str.), 1652 (C=O Str.), 2944 (C-H Str.); MS (FAB) [M + 1]+: (*m*/*z*): 381.10.

### 4.2. Molecular Docking

Docking studies of ligands ‘C1 to C5’ were conducted with Matrix Metalloproteinase-1 (MMP-1) and Matrix Metalloproteinase-2 (MMP-2). Three-dimensional structures of designed compounds were simulated within the 3D structures of MMP-1 and MMP-2. Three-dimensional structures of ligands and proteins were analyzed using ‘.pdb’ files.

Two-dimensional (2D) representations of ligands (‘C1 to C5’) were created using the ChemDraw 17.0 editor. The 2D structures were stored in .mol file format. The 2D structures were subsequently transformed into 3D structures, ensuring atomic valency was satisfied with hydrogen and optimized for the lowest energy state geometrically. Stable 3D structures were ultimately saved in .pdb format.

The crystal structures of proteins were obtained from the Protein Data Bank for MMP-1 (PDB: 3shi) and MMP-2 (PDB: 8h78). Files were obtained in PDB format. Protein structures were prepared for docking analyses. Preparation involved the removal of non-protein components from the PDB file, followed by the addition of hydrogen to satisfy valency.

Following the preparation of ‘.pdb’ files for ligand compounds and protein structures, we conducted the docking studies. Docking studies utilized AutoDock Vina software [23] to simulate the molecular interactions between ligands and proteins. AutoDockVina demonstrates superior accuracy and computational performance compared to traditional AutoDock 4.2.6 software. Each docking study yielded multiple poses for each ligand, all exhibiting negative binding affinity values measured in kilocalories per mole (kcal/mol). The pose exhibiting the highest negative binding affinity value is regarded as the optimal docked pose at the specified binding site of the corresponding target. Findings of molecular interaction in the form of docking of standard compounds and test compounds are presented in Figures S1–S14 (Supplementary Materials).

### 4.3. Nanoemulsions Formulation and Its Preparation

The oil phase of nanoemulsions was prepared by heating the ingredients (cetostearyl alcohol, stearic acid, cetomacrogol-100, lanolin, and glycerin) at 75 ± 2 °C with continuous stirring on a hot plate. The preparation of the aqueous phase involved heating purified water separately in a 2000 mL capacity beaker at 80 ± 2 °C. Methyl and propyl parabens were dissolved with intermittent stirring, and the temperature was adjusted to 75 ± 2 °C. The two phases, oil and aqueous, were combined through vigorous stirring for approximately 1 to 2 min. The synthetic derivatives were subsequently added with continuous stirring until the formation of nanoemulsions was achieved. The temperature was subsequently lowered to approximately 45 °C using a cold-water bath, and stirring was ceased. The nanoemulsions were stored in wide-mouth, airtight amber glass containers in a cool, dry location [25,26].

### 4.4. Physical Parameters of Nanoemulsions

#### 4.4.1. Color

The color was visually assessed against a dark background (Table S1).

#### 4.4.2. Odor

The assessment of the odor of a nanoemulsion entails utilizing olfactory perception to identify any fragrances or odors linked to the constituents of the nanoemulsion.

### 4.5. Physicochemical Characterization

#### 4.5.1. Particle Size and Zeta Potential Determination

Particle size and zeta potential were assessed utilizing a Malvern Zetasizer^®^ Nano-ZS 90 (Malvern Instruments, Malvern, UK). For the analysis of droplet size and zeta potential, samples were diluted 1:1000 with ultrapure water and a 1.0 mM NaCl solution prior to measurement. The tests were conducted in triplicate to verify the stability and uniformity of the prepared nanoemulsions at 25 °C.

#### 4.5.2. pH Determination

The pH was measured using a pH meter (Sky Technology India Digital Deluxe pH Meter, Model Number: STI 431(ATC), Panchkua, India). All analyses were conducted in triplicate.

#### 4.5.3. Viscosity

Viscosity refers to the measure of a fluid’s resistance to flow. It is a critical property that influences the behavior of liquids and gases in various applications.

Viscosity was measured using capillary viscometry with an Ostwald viscometer. Tests were conducted considering the flow time of samples, which were adjusted to 20 ± 0.1 °C through the capillary. All analyses were conducted in triplicate.

### 4.6. In Vitro Sun Protection Factor (SPF)

The in vitro SPF measurement techniques are a fast and admissible method for reducing the number of in vivo experiments and the risks associated with UV exposure of human subjects. The COLIPA standards were used to determine the in vitro SPF, which involves measuring the percent transmittance of a sunscreen product across the UV spectrum weighted by the erythemal weighting factors at various wavelengths [27].

$$SPF=CF\times \overset{320}{\underset{290}{\sum }}EE\left(\lambda \right)\times I(\lambda )\times Abs\left(\lambda \right)$$

where *CF* = correction factor (10), *EE* (*λ*) = erythemal action spectrum, *I* (*λ*) = solar intensity spectrum, and *Abs* (*λ*) = spectrophotometric absorbance values at wavelength *λ*.

### 4.7. In Vivo Skin Irritation Activity

#### Animals

Male albino rats (150–180 g) were procured from the Institutional Animal House at IFTM University, Lodhipur-Rajput, Moradabad, India, for the analysis of anticonvulsant activity. In polypropylene cages maintained at a temperature of 25 ± 2 °C and a relative humidity of 45–55%, the rats were maintained in reserve. The experiment was initiated after all the rats had been acclimatized to laboratory conditions for a week. These rats were provided with water ad libitum and standard animal feed. The Institutional Animal Ethics Committee (IAEC) and the Committee for Control and Supervision of Experiments on Animals (CPCSEA) of the Government of India, New Delhi, reviewed and approved the experimental protocols.

### 4.8. Study Design

Thirty-five rats were selected and randomly assigned to seven groups, with each group comprising five rats. The groups were categorized as follows: a negative control group (administered DMSO), a positive control group (administered oxybenzone), and experimental groups 1–5 (**C-1NE**, **C-2NE**, **C-3NE**, **C-4NE**, **C-5NE**; applied topically). The dorsal area of each rat was shaved the day before the experiment. Nanoemulsions were administered at a site of approximately 1 g per 1.33 cm^2^. Following a 1 h exposure to the nanoemulsions, the rats were subjected to an exotera lamp. The scoring of erythema and edema was conducted at 24 and 72 h. Skin reactions were assessed at 24 h and 72 h, focusing on erythema and edema (Table S3, Supplementary Materials). The Primary Irritation Score (SPI) was calculated for each rat. The scores for erythema and edema at 24 h and 72 h were aggregated and divided by the number of observations for the treated sites [28,29].

#### Animals Grouping and Photoprotective Analysis by In Vivo Method

The in vivo SPFs were assessed using the FDA methodology. This study utilized 42 albino rats of the Wistar strain, each weighing between 185 and 230 g. To assess the photoprotective effects of the formulations, six groups of six animals each were organized as follows:

Group 1: UV Radiation and Nanoemulsion Base;
Group 2: Ultraviolet Radiation and Oxybenzone;
Group 3: Ultraviolet Radiation and **C-1NE**;
Group 4: Ultraviolet Radiation and **C-2NE**;
Group 5: Ultraviolet Radiation and **C-3NE**;
Group 6: Ultraviolet Radiation and **C-4NE**;
Group 7: Ultraviolet Radiation and **C-5NE**.

The current study utilized previously calibrated ultraviolet range lamps from Solar Light Co., Chennai, India. The UV dose increase for each instance was 25%, and the total test area measured 25 cm^2^.

The SPF value represents the UV energy necessary to induce a minimum erythemal dose (MED), or redness, on protected skin, divided by the UV energy needed to produce a MED on unprotected skin.

The sample quantity was measured and uniformly administered to the skin at a density of 2.0 mg/cm^2^. The interval between the application of the formulation and the irradiation was 15 min. A dosage of 2.5 mg cm^−2^ was administered to animals in groups 2, 3, 4, 5, and 6. The minimal erythemal dose (MED) was assessed visually following 22–24 h of UV exposure. The SPF values of all formulations were calculated based on the UVR dose necessary to induce MED on protected skin compared to the UVR dose required to induce MED on unprotected skin [30,31,32].

## 5. Conclusions

Five chalcone derivatives were efficiently synthesized and subsequently characterized employing spectroscopic techniques. The synthesized compounds **C-5**, **C-4**, and **C-3** displayed the highest binding affinity for MMP-1, whereas **C-3** and **C-4** exhibited substantial binding affinities for MMP-2. In both in vitro and in vivo SPF investigations, **C-4NE** and **C-3NE** exhibited the highest SPF values. This further indicates the potential for formulation development utilizing C4 and C3 compounds, as the resulting formulation is expected to mitigate the detrimental effects of ultraviolet radiation and sunburn. Further, this will protect the dermis via MMP pathways. Based upon previous work and present findings, it may be strongly recommended that these chalcone derivatives may serve as a potential lead for sun protection factor (SPF) activity, which may subsequently be structurally modified to yield compounds exhibiting enhanced SPF activity and subsequently effective pharmaceutical formulation may be developed.

## Figures and Tables

**Table 1:** Physical characterization data of synthesized compounds.

S. No.	Compound Code	Structure of Compound	Molecular Formula	Molecular Weight	% Yield	Melting Point (MP) (^o^C)	R_f_ Value	% Purity
1.	**I-1**	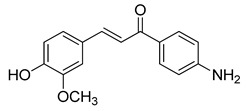	C_16_H_15_NO_3_	269.30	60.02	65–68	0.50	71.29
2.	**I-2**	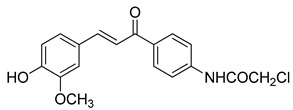	C_18_H_16_ClNO_4_	345.78	80.12	110–114	0.36	73.22
3.	**C-1**	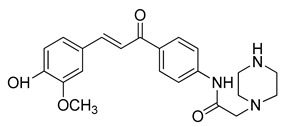	C_22_H_25_N_3_O_4_	395.45	72.09	120–122	0.60	68.89
4.	**C-2**	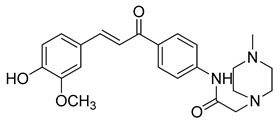	C_23_H_27_N_3_O_4_	409.51	62.12	75–78	0.44	76.65
5.	**C-3**	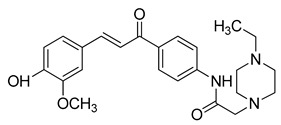	C_24_H_29_N_3_O_4_	423.50	58.16	130–132	0.62	68.99
6.	**C-4**	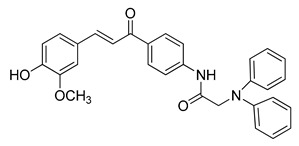	C_30_H_26_N_2_O_4_	478.19	66.26	90–94	0.88	77.23
7.	**C-5**	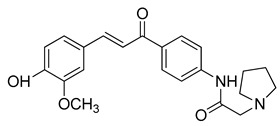	C_22_H_24_N_2_O_4_	380.44	70.32	150–152	0.74	72.79

**Table 2:** Binding affinities of synthesized compounds **C-1** to **C-5** with MMP-1 and MMP-2.

Compounds	Binding Affinity (Kcal/mol) for MMP-1	Binding Affinity (Kcal/mol) for MMP-2
**C-1**	−7.8	−9.6
**C-2**	−8.3	−9.7
**C-3**	−7.7	−9.9
**C-4**	−8.5	−9.7
**C-5**	−8.2	−9.1
Oxybenzone	−6.6	−8.6
Vanillin	−6.4	−5.9

Formulation of O/W nanoemulsion and characterization.

**Table 3:** Ingredients used in the preparation of *nanoemulsion*.

S. No.	Ingredients	Uses	Components (%*w*/*w*)
1	Cetostearyl alcohol	Emulsifier	35
2	Stearic acid	Emollient, Co-emulsifier	40
3	Cetomacrogol-100	Emulsifier	9
4	Lanolin	Emollient	50
5	Glycerin	Humectant	156.6
6	Methyl paraben	Preservative	4
7	Propyl paraben	Preservative	0.4
8	**C-1**/**C-2**/**C-3**/**C-4**/**C-5**	Active ingredient	10
9	Distilled water	Vehicle	695

**Table 4:** In vitro SPF value of nanoemulsions measured under different wavelengths.

S.No	Wavelength (λ nm)	EE × I (Normalized)	Abs × EE × I Oxybenzone	Abs × EE × I C1-NE	Abs × EE × I C2-NE	Abs × EE × I C3-NE	Abs × EE × I C4-NE	Abs × EE × I C5-NE
1	290	0.0150	0.0117	0.0303	0.0032	0.0075	0.0101	0.0016
2	295	0.0817	0.0712	0.0012	0.0131	0.0580	0.0661	0.0100
3	300	0.2874	0.2730	0.0899	0.0888	0.1968	0.2805	0.0704
4	305	0.3278	0.2914	0.0668	0.0708	0.1858	0.2871	0.0691
5	310	0.1864	0.1720	0.0469	0.0534	0.1524	0.1444	0.0657
6	315	0.0837	0.0779	0.0193	0.0208	0.0754	0.0742	0.0103
7	320	0.0180	0.0174	0.0038	0.0038	0.0117	0.0174	0.0037
SPF			9.146	2.582	2.539	6.876	8.798	2.308

Where = EE (λ) = erythemal action spectrum, I (λ) = solar intensity spectrum, and Abs (λ) = spectrophotometric absorbance values at wavelength λ. The experiment was performed in triplicate, and mean data are presented.

## Data Availability

Data will be made available on demand.

## Supplementary Materials

The following supporting information can be downloaded at https://www.mdpi.com/article/10.3390/molecules30051057/s1, Figure S1: Molecular interaction of Vanillin with PDB: 3shi of MMP-1. (A) and (B) are 3D view, while (C) is showing 2D view of molecular interaction; Figure S2: Molecular interaction of oxybenzone with PDB: 3shi of MMP-1. (A) and (B) are 3D view, while (C) is showing 2D view of molecular interaction, Figure S3: Molecular interaction of C1 with PDB: 3shi of MMP-1. Figure S4: Molecular interaction of C2 with PDB: 3shi of MMP1. Figure S5: Molecular interaction of C3 with PDB: 3shi of MMP1. Figure S6: Molecular interaction of C4 with PDB: 3shi of MMP1. Figure S7: Molecular interaction of C5 with PDB: 3shi of MMP1. Figure S8: Molecular interaction of Vanillin with PDB: 3shi of MMP-2. (A) and (B) are 3D view, while (C) is showing 2D view of molecular interaction; Figure S9: Molecular interaction of oxybenzone with PDB: 3shi of MMP1. (A) and (B) are 3D view, while (C) is showing 2D view of molecular interaction, Figure S10: Molecular interaction of C1 with PDB: 3shi of MMP-2. Figure S11: Molecular interaction of C2 with PDB: 3shi of MMP-2. Figure S12: Molecular interaction of C3 with PDB: 3shi of MMP2. Figure S13: Molecular interaction of C4 with PDB: 3shi of MMP2. Figure S14: Molecular interaction of C5 with PDB: 3shi of MMP-2. Table S1: Physical Parameters of Nanoemulsions; Table S2: Physicochemical characteristics of Nanoemulsions; Table S3: Score for skin reaction; Table S4: In vivo SPF values; Table S5: Erythema Scores with diameter range

## Abbreviations

The following abbreviations are used in this manuscript:

MMP	Matrix Metalloproteinases
SPF	Sun Protection Factor
UV	Ultraviolet
