# Supplementary material for: Synthesis, Characterization, and Docking Studies of Some New Chalcone Derivatives to Alleviate Skin Damage Due to UV Light
Source: Molecules. 2025 Feb 25;30(5):1057. doi: 10.3390/molecules30051057 (PMC11901719; doi:10.3390/molecules30051057)
Supplement: Supplementary file 1 [file molecules-30-01057-s001.zip › ARRIVE Compliance Questionnaire.pdf]

The ARRIVE Essential 10: Compliance Questionnaire

Use this questionnaire to evaluate how well a manuscript complies with the ARRIVE Essential 10. It can be applied to any manuscript describing comparative experiments in living animals, by assessors such as journal staff, editors, or peer reviewers.

Item		Question(s)	Answers
1	Study Design	Are all experimental and control groups clearly identified?	☒ Yes, for at least one experiment<br>☐ No
		Is the experimental unit (e.g. an animal, litter or cage of animals) clearly identified?	☒ Yes, for at least one experiment<br>☐ No
2	Sample Size	Is the exact number of experimental units in each group at the start of the study provided (e.g. in the format '*n*=')?	☒ Yes, for at least one experiment<br>☐ No
		Is the method by which the sample size was chosen explained?	☒ Yes, for at least one experiment<br>☐ No
3	Inclusion & Exclusion Criteria	Are the criteria used for including and excluding animals, experimental units, or data points provided?	☐ Yes, for at least one experiment<br>☒ No
		Are any exclusions of animals, experimental units, or data points reported, or is there a statement indicating that there were no exclusions?	☐ Yes, for at least one analysis<br>☐ No
4	Randomisation	Is the method by which experimental units were allocated to control and treatment groups described?	☒ Yes, for at least one experiment<br>☐ No
5	Blinding	Is it clear whether researchers were aware of, or blinded to, the group allocation at any stage of the experiment or data analysis?	☒ Yes, for at least one experiment<br>☐ No
6	Outcome Measures	For all experimental outcomes presented, are details provided of exactly what parameter was measured?	☒ Yes, for at least one experiment<br>☐ No
7	Statistical Methods	Is the statistical approach used to analyse each outcome detailed?	☒ Yes, for at least one analysis<br>☐ No
		Is there a description of any methods used to assess whether data met statistical assumptions?	☐ Yes, for at least one analysis<br>☐ No<br>☒ Not applicable
8	Experimental Animals	Are all species of animal used specified?	☒ Yes, for at least one experiment<br>☐ No
		Is the sex of the animals specified?	☒ Yes, for at least one experiment<br>☐ No<br>☐ Not applicable to species
		Is at least one of age, weight or developmental stage of the animals specified?	☒ Yes, for at least one experiment<br>☐ No
9	Experimental Procedures	Are both the timing and frequency with which procedures took place specified?	☒ Yes, for at least one experiment<br>☐ No
		Are details of acclimatisation periods to experimental locations provided?	☒ Yes, for at least one experiment<br>☐ No
10	Results	Are descriptive statistics for each experimental group provided, with a measure of variability (e.g. mean and SD, or median and range)?	☐ Yes, for at least one experiment<br>☐ No<br>☒ Not applicable to the type of data collected
		Is the effect size and confidence interval provided?	☐ Yes, for at least one experiment<br>☐ No<br>☒ Not applicable to the type of analysis used
