# Supplementary material for: Synthesis, Characterization, and Docking Studies of Some New Chalcone Derivatives to Alleviate Skin Damage Due to UV Light
Source: Molecules. 2025 Feb 25;30(5):1057. doi: 10.3390/molecules30051057 (PMC11901719; doi:10.3390/molecules30051057)
Supplement: Supplementary file 1 [file molecules-30-01057-s001.zip › Spectra IR_NMR.pdf]

IR Spectra

(I-1)

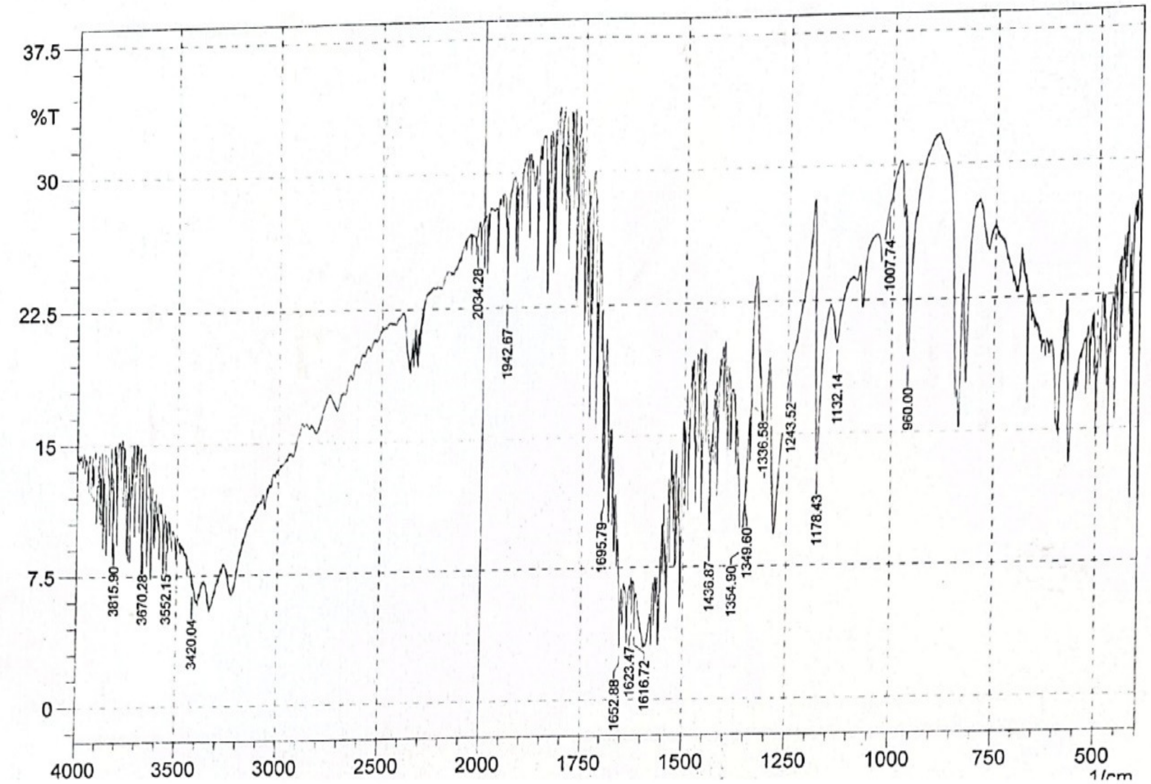

I-2

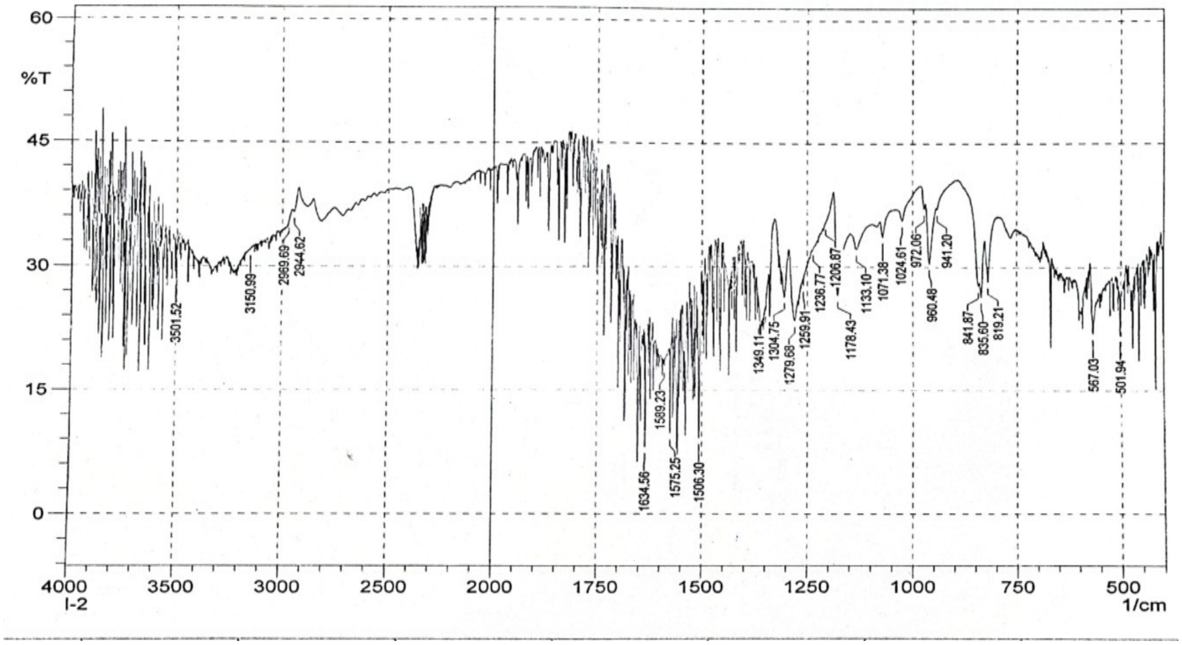

C-1

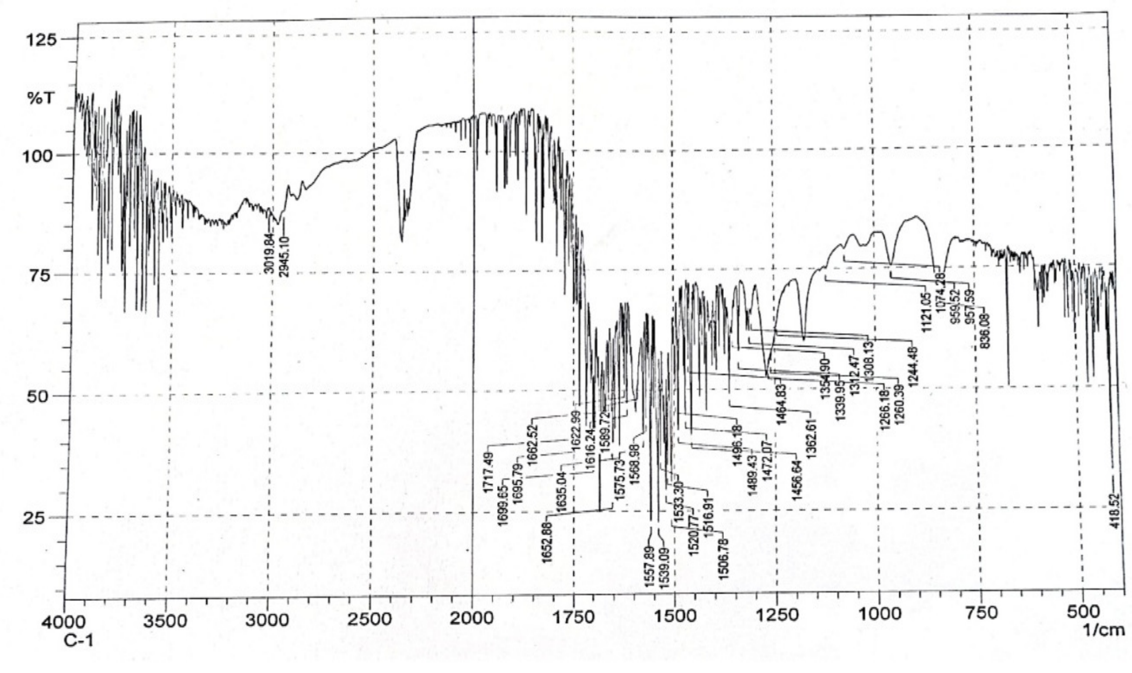
125
%T
100
75
50
25
4000
3500
3000
2500
2000
1750
1500
1250
1000
750
500
1/cm
C-1
3019.84
2945.10
1717.49
1699.65
1695.79
1662.52
1652.88
1635.04
1622.99
1616.24
1589.72
1575.73
1568.98
1557.89
1539.09
1533.30
1520.77
1516.91
1506.78
1496.18
1489.43
1472.07
1464.83
1456.64
1362.61
1354.90
1339.95
1312.47
1308.13
1266.18
1260.39
1244.48
1121.05
1074.28
959.52
957.59
836.08
418.52

C-2

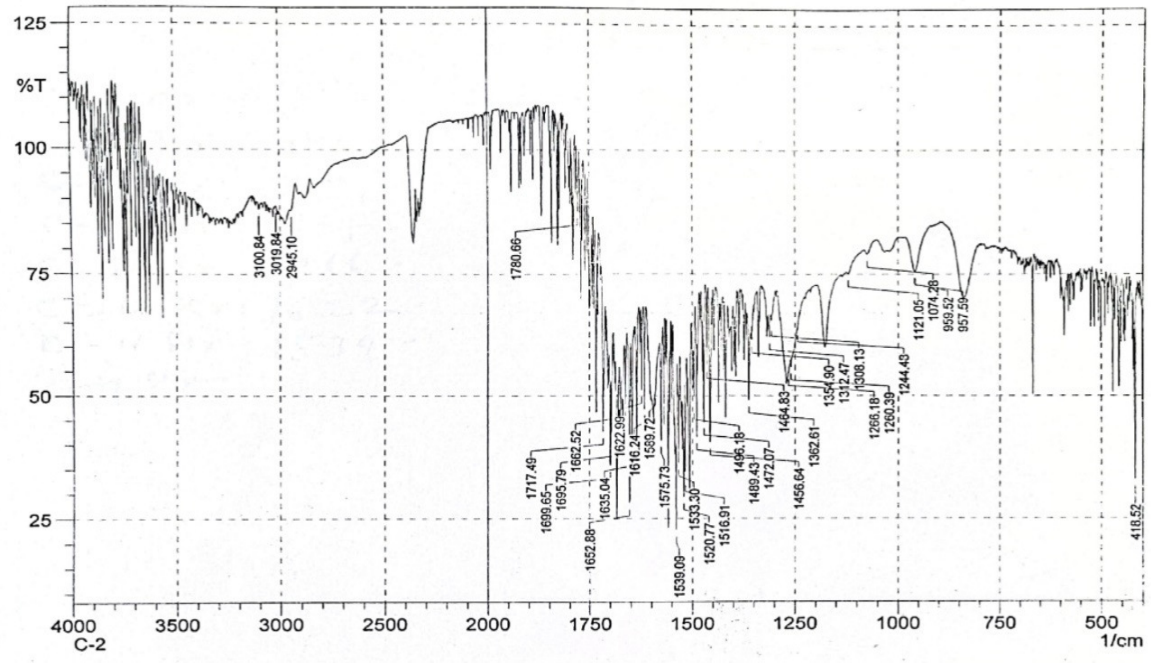
125
%T
100
75
50
25
4000
3500
3000
2500
2000
1750
1500
1250
1000
750
500
1/cm
C-2
3100.84
3019.84
2945.10
1780.66
1717.49
1699.65
1695.79
1662.52
1652.88
1635.04
1622.99
1616.24
1589.72
1575.73
1539.09
1533.30
1520.77
1516.91
1496.18
1489.43
1472.07
1464.83
1456.64
1362.61
1354.90
1312.47
1308.13
1266.18
1260.39
1244.43
1121.05
1074.28
959.52
957.59
418.52

C-3

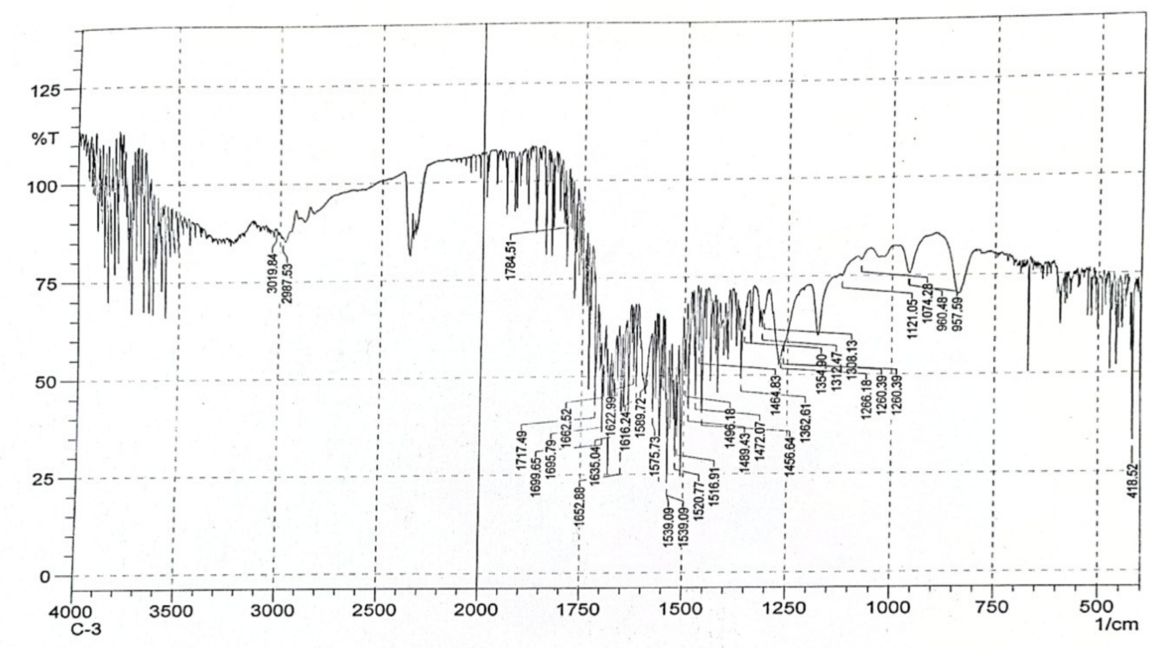
%T
125
100
75
50
25
0
4000
3500
3000
2500
2000
1750
1500
1250
1000
750
500
1/cm
C-3
3019.84
2987.53
1784.51
1717.49
1699.65
1695.79
1662.52
1652.88
1635.04
1622.99
1616.24
1589.72
1575.73
1539.09
1539.09
1520.77
1516.91
1496.18
1489.43
1472.07
1464.83
1456.64
1362.61
1354.90
1312.47
1308.13
1266.18
1260.39
1260.39
1121.05
1074.28
960.48
957.59
418.52

C-4

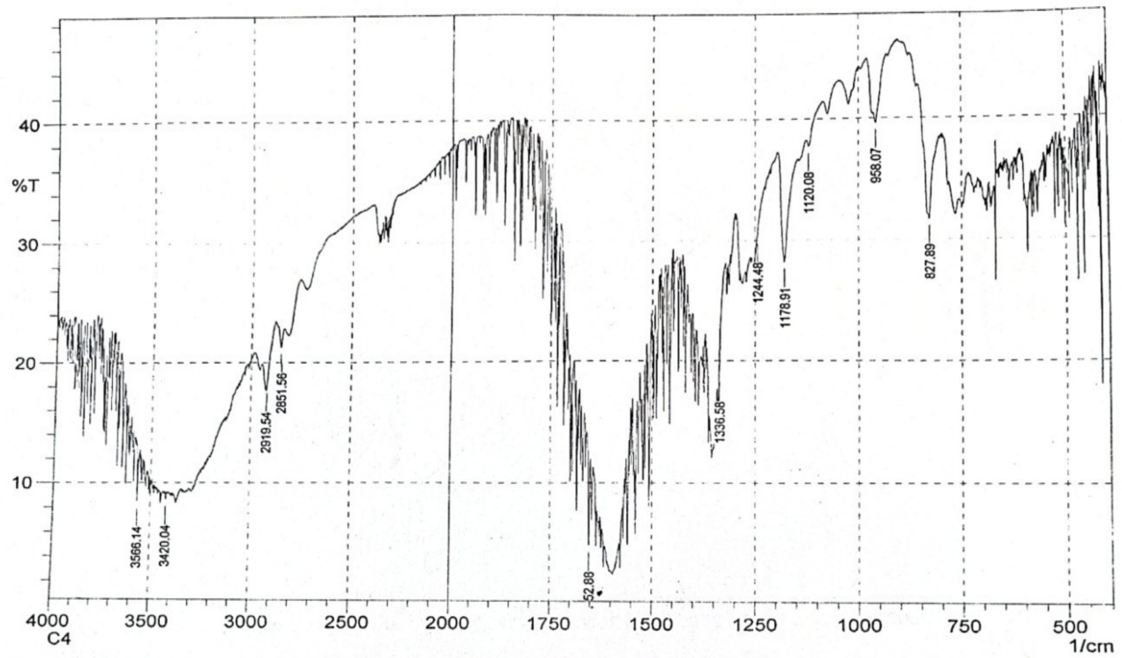
%T
40
30
20
10
4000
3500
3000
2500
2000
1750
1500
1250
1000
750
500
1/cm
C4
3566.14
3420.04
2919.54
2851.56
1336.58
1244.46
1178.91
1120.08
958.07
827.89

C-5

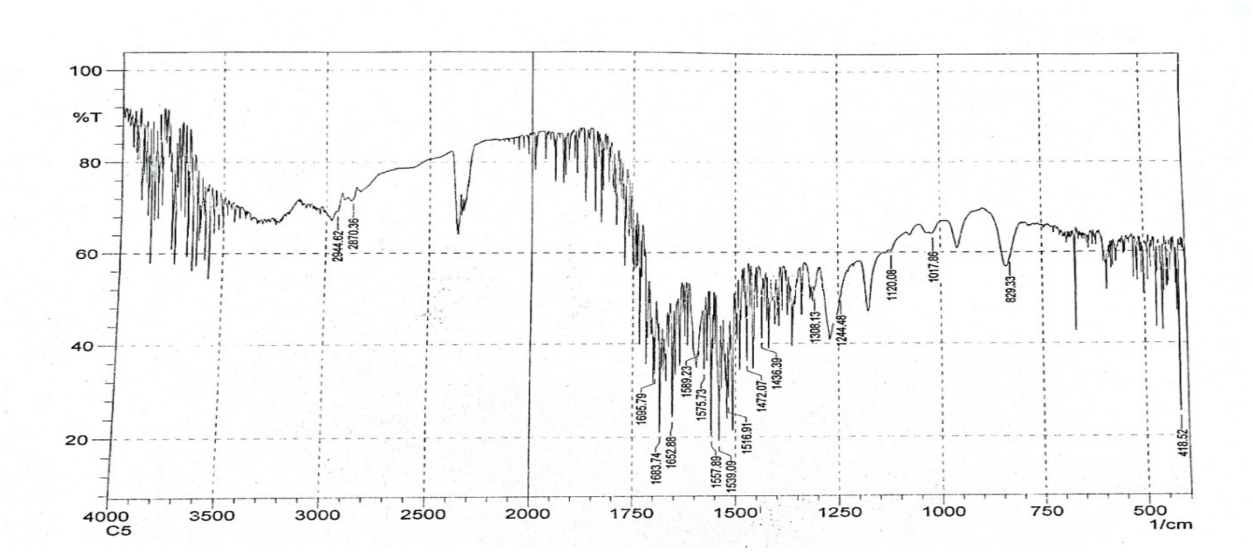
100
%T
80
60
40
20
4000
3500
3000
2500
2000
1750
1500
1250
1000
750
500
1/cm
C5
2944.62
2870.36
1695.79
1683.74
1652.88
1589.23
1575.73
1557.89
1539.09
1516.91
1472.07
1436.39
1308.13
1244.48
1120.08
1017.86
829.33
418.52

1HNMR

I-1

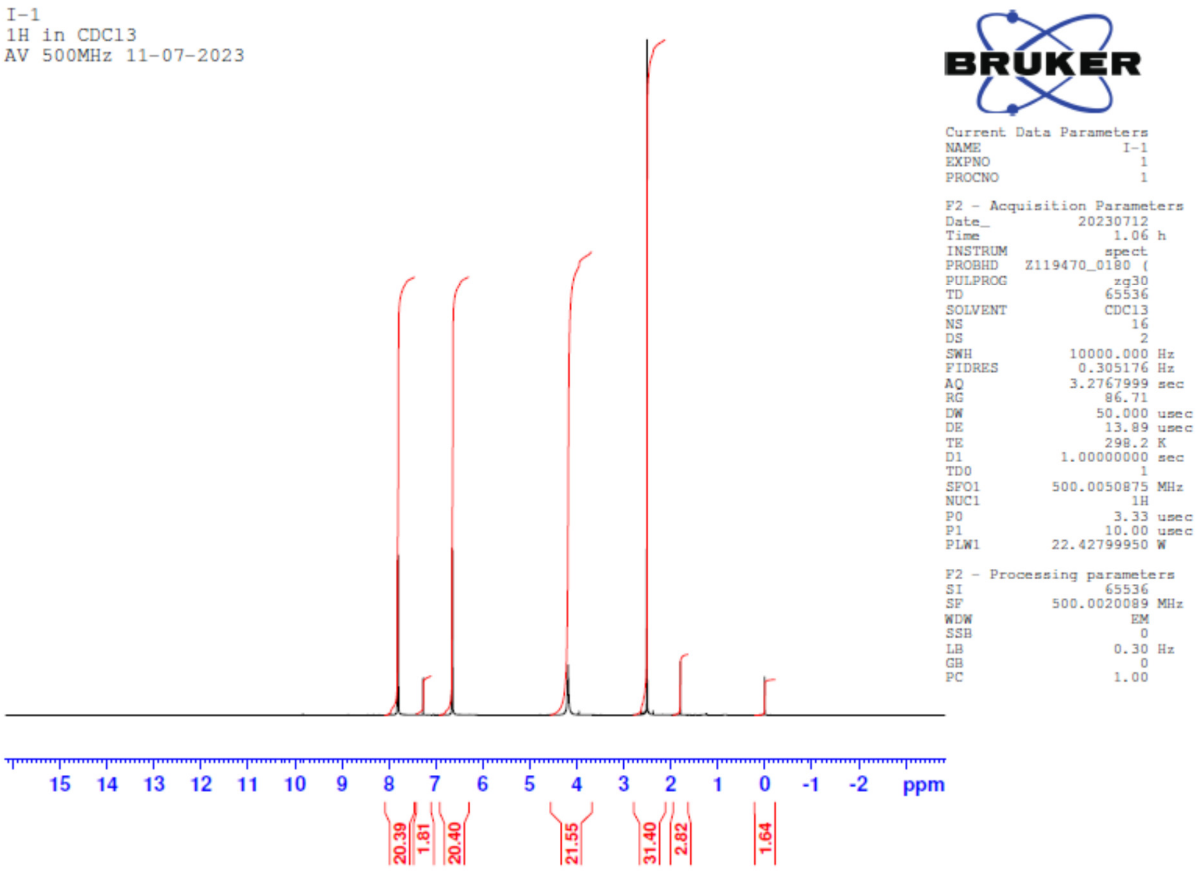
I-1
1H in CDCl3
AV 500MHz 11-07-2023
BRUKER
Current Data Parameters
NAME I-1
EXPNO 1
PROCNO 1
F2 - Acquisition Parameters
Date_ 20230712
Time 1.06 h
INSTRUM spect
PROBHD Z119470_0180 (
PULPROG zg30
TD 65536
SOLVENT CDCl3
NS 16
DS 2
SWH 10000.000 Hz
FIDRES 0.305176 Hz
AQ 3.2767999 sec
RG 86.71
DW 50.000 usec
DE 13.89 usec
TE 298.2 K
D1 1.00000000 sec
TD0 1
SFO1 500.0050875 MHz
NUC1 1H
P0 3.33 usec
P1 10.00 usec
PLW1 22.42799950 W
F2 - Processing parameters
SI 65536
SF 500.0020089 MHz
WDW EM
SSB 0
LB 0.30 Hz
GB 0
PC 1.00
15 14 13 12 11 10 9 8 7 6 5 4 3 2 1 0 -1 -2 ppm
20.39
1.81
20.40
21.55
31.40
2.82
1.64

I-2

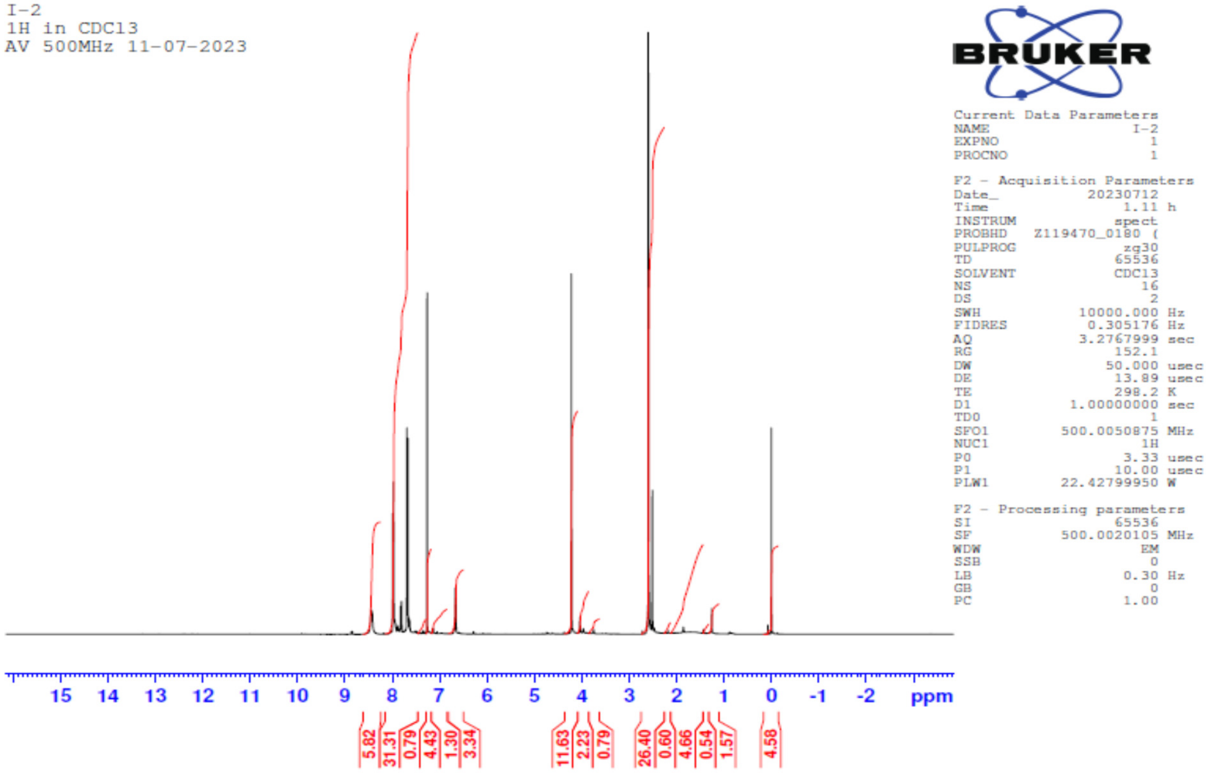
I-2
1H in CDCl3
AV 500MHz 11-07-2023
BRUKER
Current Data Parameters
NAME I-2
EXPNO 1
PROCNO 1
F2 - Acquisition Parameters
Date_ 20230712
Time 1.11 h
INSTRUM spect
PROBHD Z119470_0180 (
PULPROG zg30
TD 65536
SOLVENT CDCl3
NS 16
DS 2
SWH 10000.000 Hz
FIDRES 0.305176 Hz
AQ 3.2767999 sec
RG 152.1
DW 50.000 usec
DE 13.89 usec
TE 298.2 K
D1 1.00000000 sec
TD0 1
SFO1 500.0050875 MHz
NUC1 1H
P0 3.33 usec
P1 10.00 usec
PLW1 22.42799950 W
F2 - Processing parameters
SI 65536
SF 500.0020105 MHz
WDW EM
SSB 0
LB 0.30 Hz
GB 0
PC 1.00
ppm

C-1

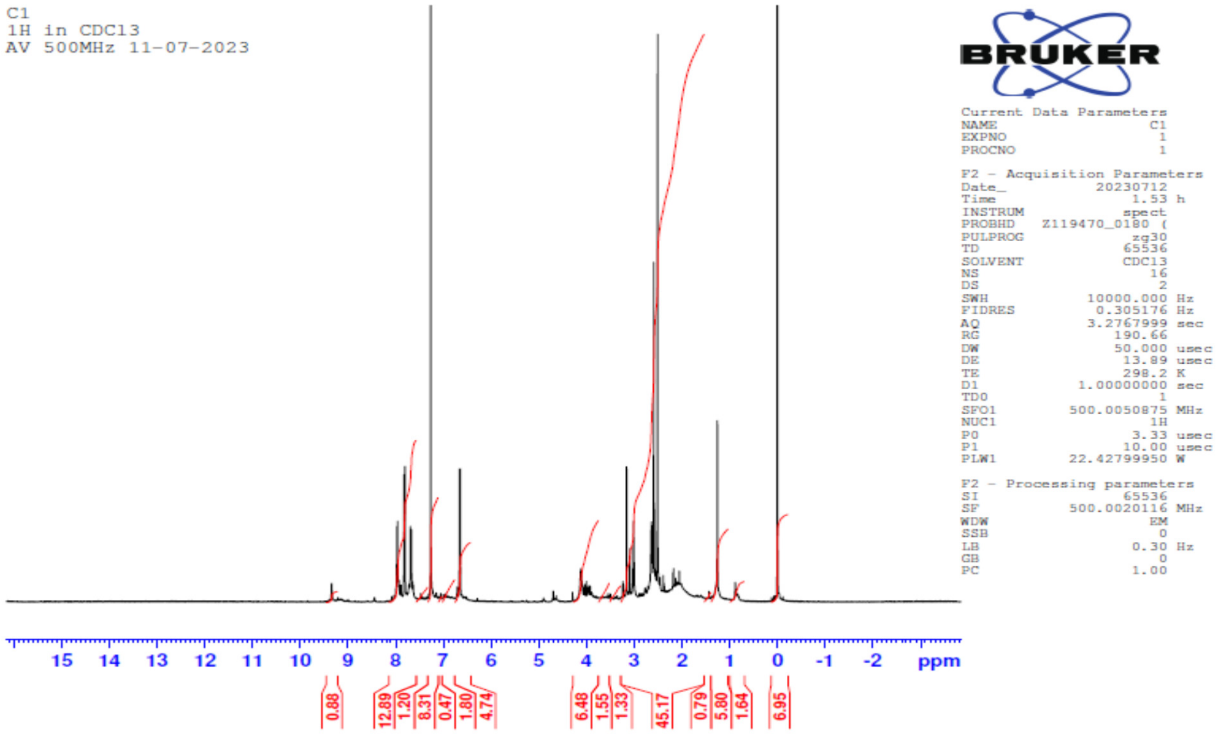
C1
1H in CDCl3
AV 500MHz 11-07-2023
BRUKER
Current Data Parameters
NAME C1
EXPNO 1
PROCNO 1
F2 - Acquisition Parameters
Date_ 20230712
Time 1.53 h
INSTRUM spect
PROBHD Z119470_0180 (
PULPROG zg30
TD 65536
SOLVENT CDCl3
NS 16
DS 2
SWH 10000.000 Hz
FIDRES 0.305176 Hz
AQ 3.2767999 sec
RG 190.66
DW 50.000 usec
DE 13.89 usec
TE 298.2 K
D1 1.00000000 sec
TD0 1
SFO1 500.0050875 MHz
NUC1 1H
P0 3.33 usec
P1 10.00 usec
PLW1 22.42799950 W
F2 - Processing parameters
SI 65536
SF 500.0020116 MHz
WDW EM
SSB 0
LB 0.30 Hz
GB 0
PC 1.00
ppm

C-2

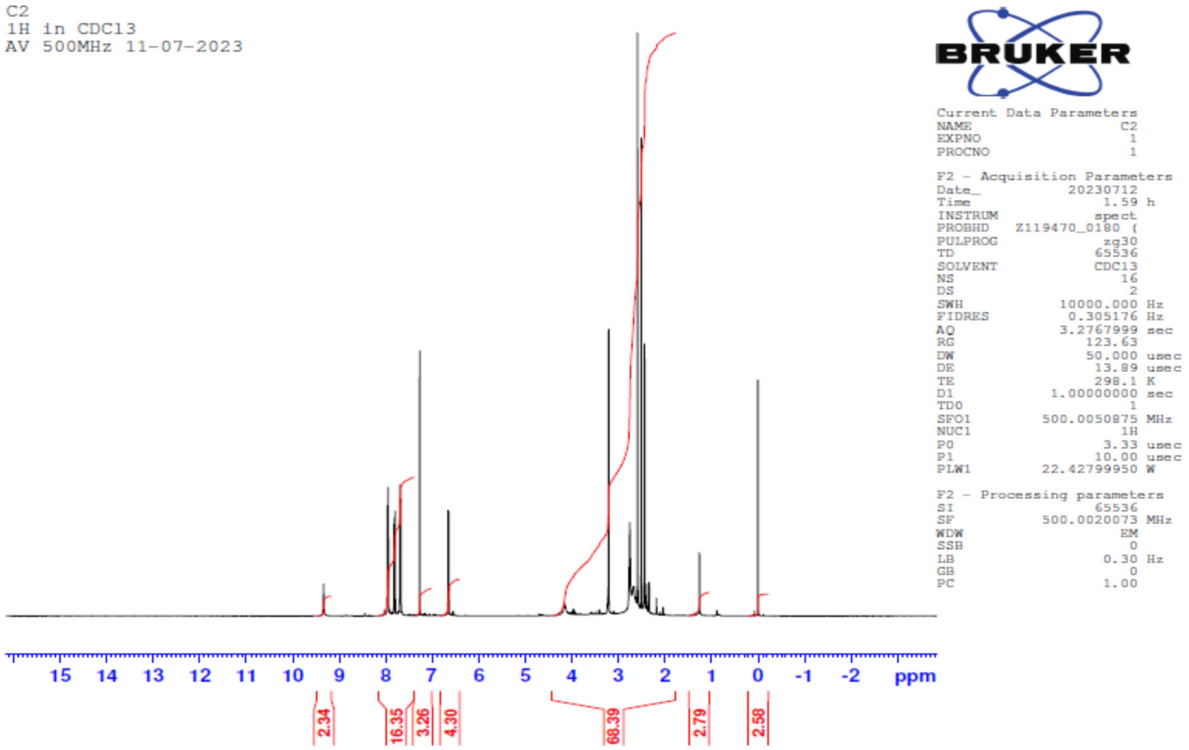
C2
1H in CDCl3
AV 500MHz 11-07-2023
BRUKER
Current Data Parameters
NAME C2
EXPNO 1
PROCNO 1
F2 - Acquisition Parameters
Date_ 20230712
Time 1.59 h
INSTRUM spect
PROBHD Z119470_0180 (
PULPROG zg30
TD 65536
SOLVENT CDCl3
NS 16
DS 2
SWH 10000.000 Hz
FIDRES 0.305176 Hz
AQ 3.2767999 sec
RG 123.63
DW 50.000 usec
DE 13.89 usec
TE 298.1 K
D1 1.00000000 sec
TD0 1
SFO1 500.0050875 MHz
NUC1 1H
P0 3.33 usec
P1 10.00 usec
PLW1 22.42799950 W
F2 - Processing parameters
SI 65536
SF 500.0020073 MHz
WDW EM
SSB 0
LB 0.30 Hz
GB 0
PC 1.00
15 14 13 12 11 10 9 8 7 6 5 4 3 2 1 0 -1 -2 ppm
2.34
16.35
3.26
4.30
68.39
2.79
2.58

C-3

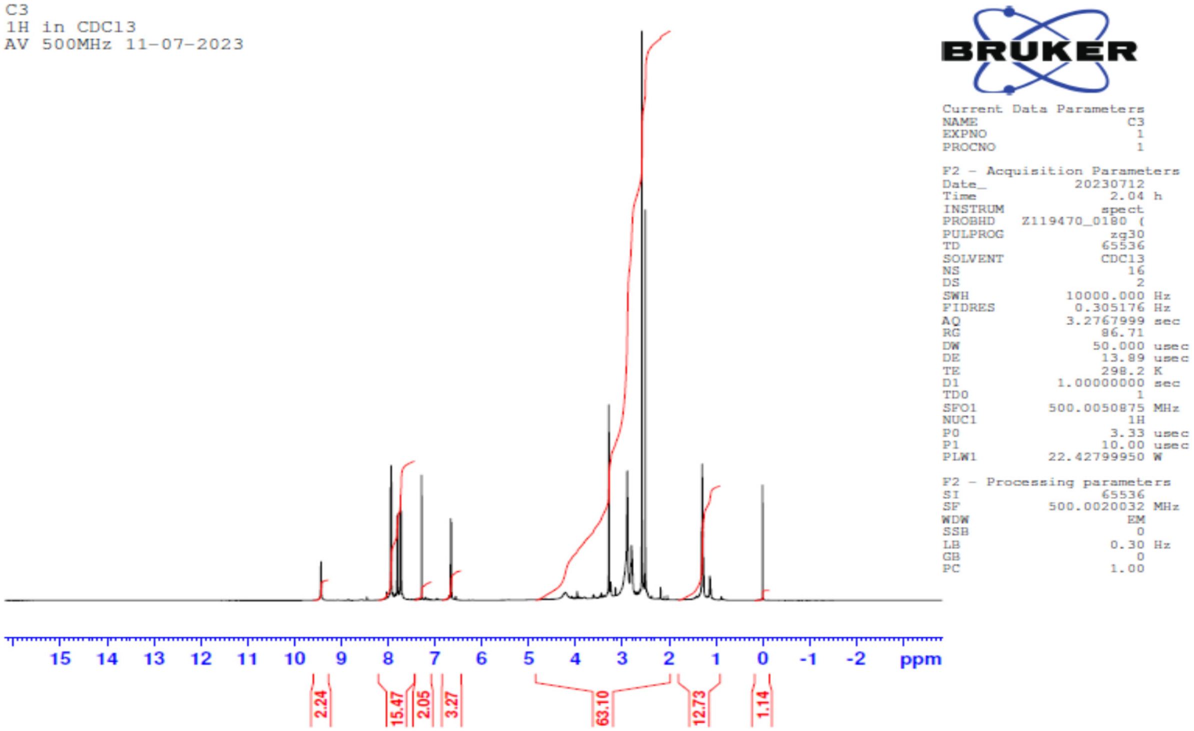
C3
1H in CDCl3
AV 500MHz 11-07-2023
BRUKER
Current Data Parameters
NAME C3
EXPNO 1
PROCNO 1
F2 - Acquisition Parameters
Date_ 20230712
Time 2.04 h
INSTRUM spect
PROBHD Z119470_0180 (
PULPROG zg30
TD 65536
SOLVENT CDCl3
NS 16
DS 2
SWH 10000.000 Hz
FIDRES 0.305176 Hz
AQ 3.2767999 sec
RG 86.71
DW 50.000 usec
DE 13.89 usec
TE 298.2 K
D1 1.00000000 sec
TD0 1
SFO1 500.0050875 MHz
NUC1 1H
P0 3.33 usec
P1 10.00 usec
PLW1 22.42799950 W
F2 - Processing parameters
SI 65536
SF 500.0020032 MHz
WDW EM
SSB 0
LB 0.30 Hz
GB 0
PC 1.00
15 14 13 12 11 10 9 8 7 6 5 4 3 2 1 0 -1 -2 ppm
2.24
15.47
2.05
3.27
63.10
12.73
1.14

C-4

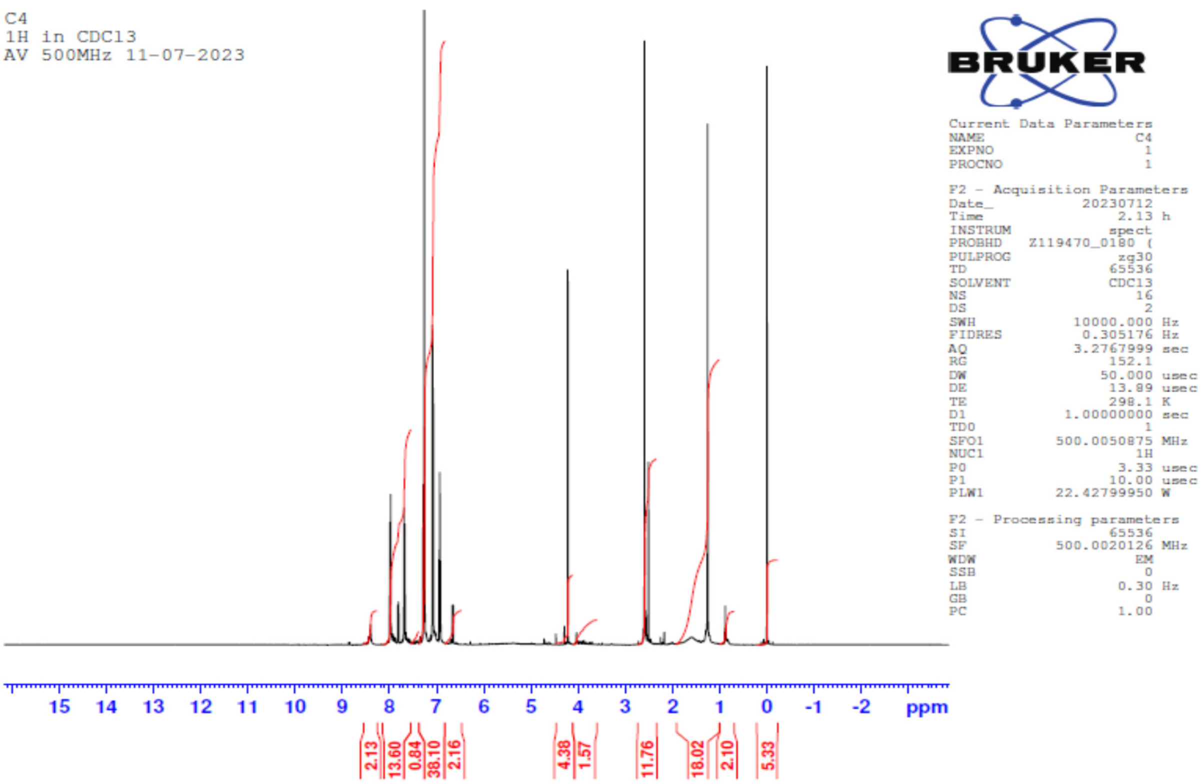
C4
1H in CDCl3
AV 500MHz 11-07-2023
BRUKER
Current Data Parameters
NAME C4
EXPNO 1
PROCNO 1
F2 - Acquisition Parameters
Date_ 20230712
Time 2.13 h
INSTRUM spect
PROBHD Z119470_0180 (
PULPROG zg30
TD 65536
SOLVENT CDCl3
NS 16
DS 2
SWH 10000.000 Hz
FIDRES 0.305176 Hz
AQ 3.2767999 sec
RG 152.1
DW 50.000 usec
DE 13.89 usec
TE 298.1 K
D1 1.00000000 sec
TD0 1
SFO1 500.0050875 MHz
NUC1 1H
P0 3.33 usec
P1 10.00 usec
PLW1 22.42799950 W
F2 - Processing parameters
SI 65536
SF 500.0020126 MHz
WDW EM
SSB 0
LB 0.30 Hz
GB 0
PC 1.00
15 14 13 12 11 10 9 8 7 6 5 4 3 2 1 0 -1 -2 ppm
2.13 13.60 0.84 38.10 2.16 4.38 1.57 11.76 18.02 2.10 5.33

C-5

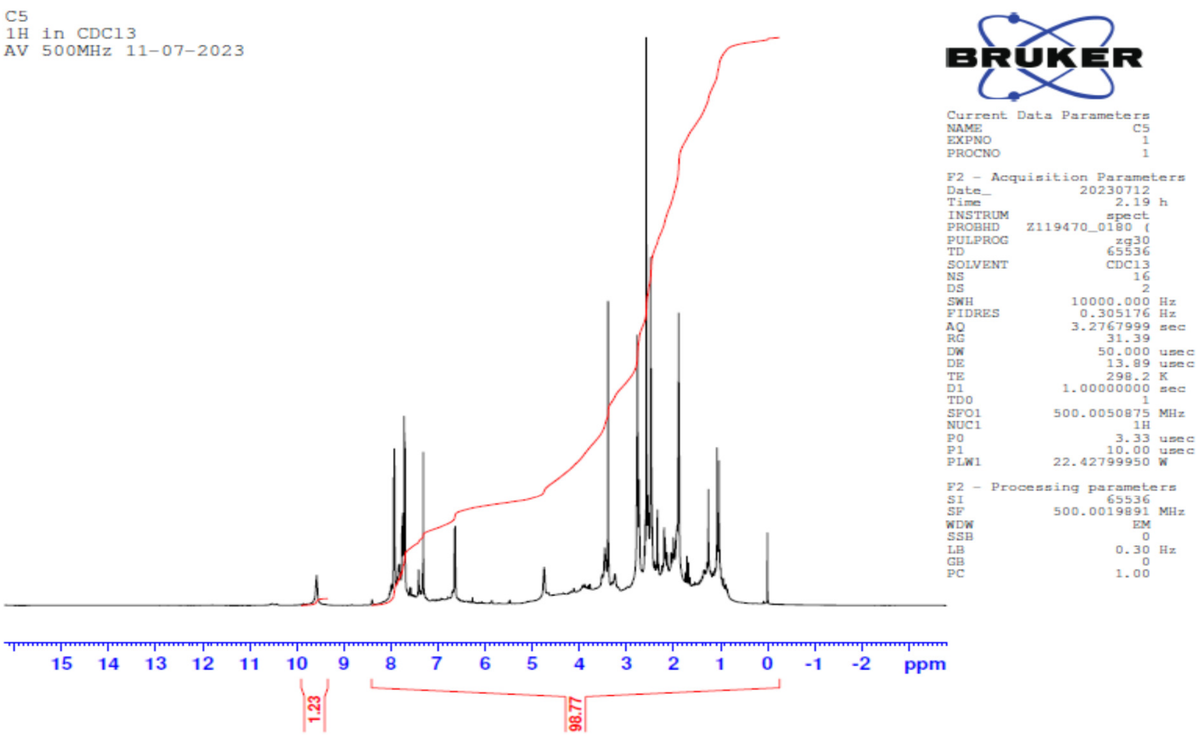
C5
1H in CDCl3
AV 500MHz 11-07-2023
BRUKER
Current Data Parameters
NAME C5
EXPNO 1
PROCNO 1
F2 - Acquisition Parameters
Date_ 20230712
Time 2.19 h
INSTRUM spect
PROBHD Z119470_0180 (
PULPROG zg30
TD 65536
SOLVENT CDCl3
NS 16
DS 2
SWH 10000.000 Hz
FIDRES 0.305176 Hz
AQ 3.2767999 sec
RG 31.39
DW 50.000 usec
DE 13.89 usec
TE 298.2 K
D1 1.00000000 sec
TD0 1
SFO1 500.0050875 MHz
NUC1 1H
P0 3.33 usec
P1 10.00 usec
PLW1 22.42799950 W
F2 - Processing parameters
SI 65536
SF 500.0019891 MHz
WDW EM
SSB 0
LB 0.30 Hz
GB 0
PC 1.00
15 14 13 12 11 10 9 8 7 6 5 4 3 2 1 0 -1 -2 ppm
1.23 98.77

13CNMR

I-1

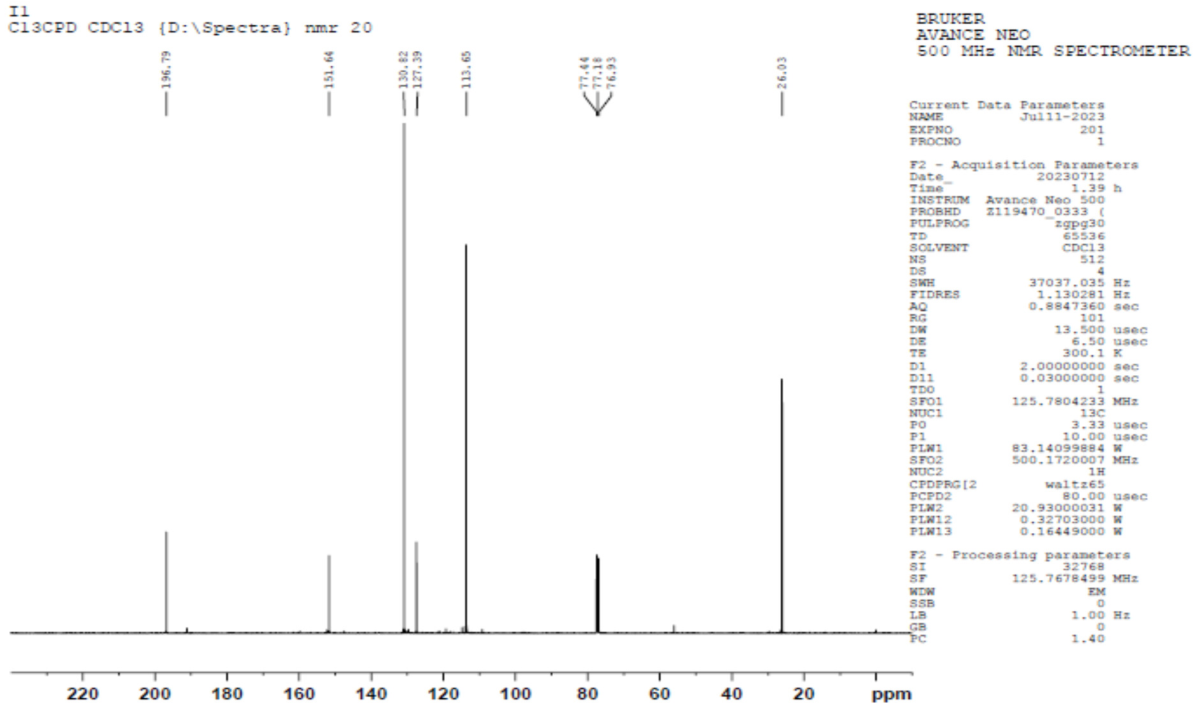
I1
C13CPD CDCl3 {D:\Spectra} nmr 20
BRUKER
AVANCE NEO
500 MHz NMR SPECTROMETER
196.79
151.64
130.82
127.39
113.65
77.44
77.18
76.93
26.03
ppm

I-2

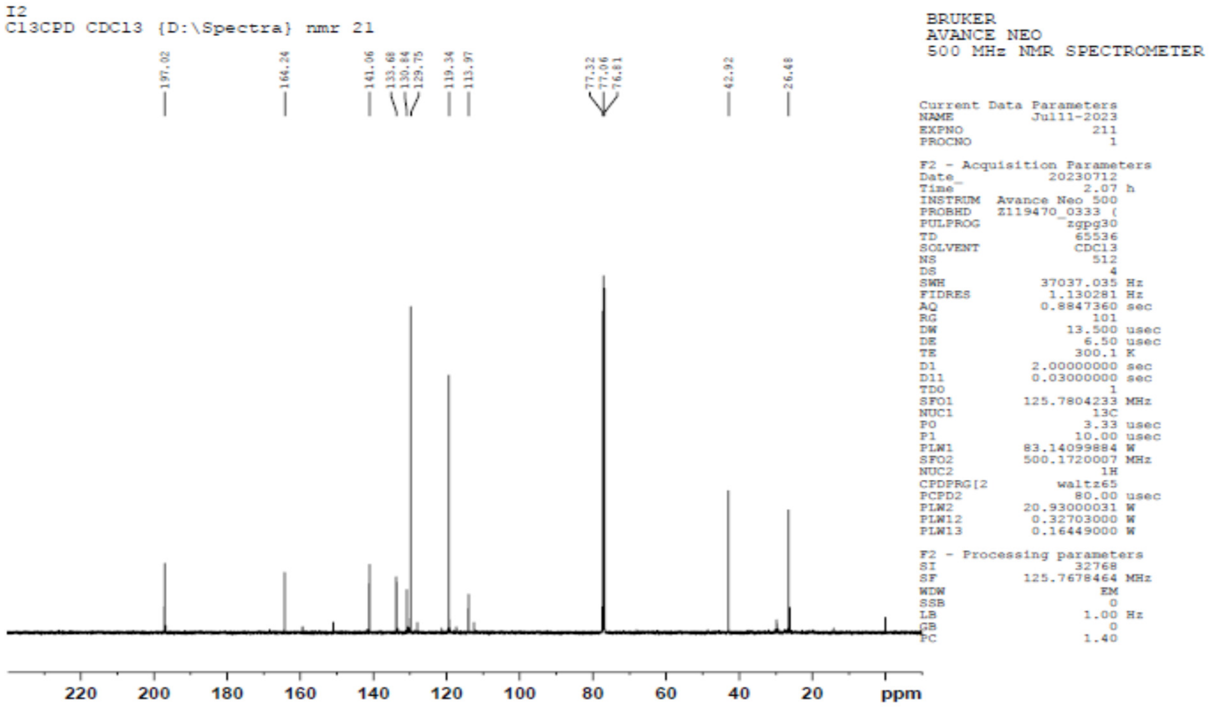
I2
C13CPD CDCl3 {D:\Spectra} nmr 21
BRUKER
AVANCE NEO
500 MHz NMR SPECTROMETER
197.02
164.24
141.06
133.68
130.84
129.75
119.34
113.97
77.32
77.06
76.81
42.92
26.48
ppm

C-1

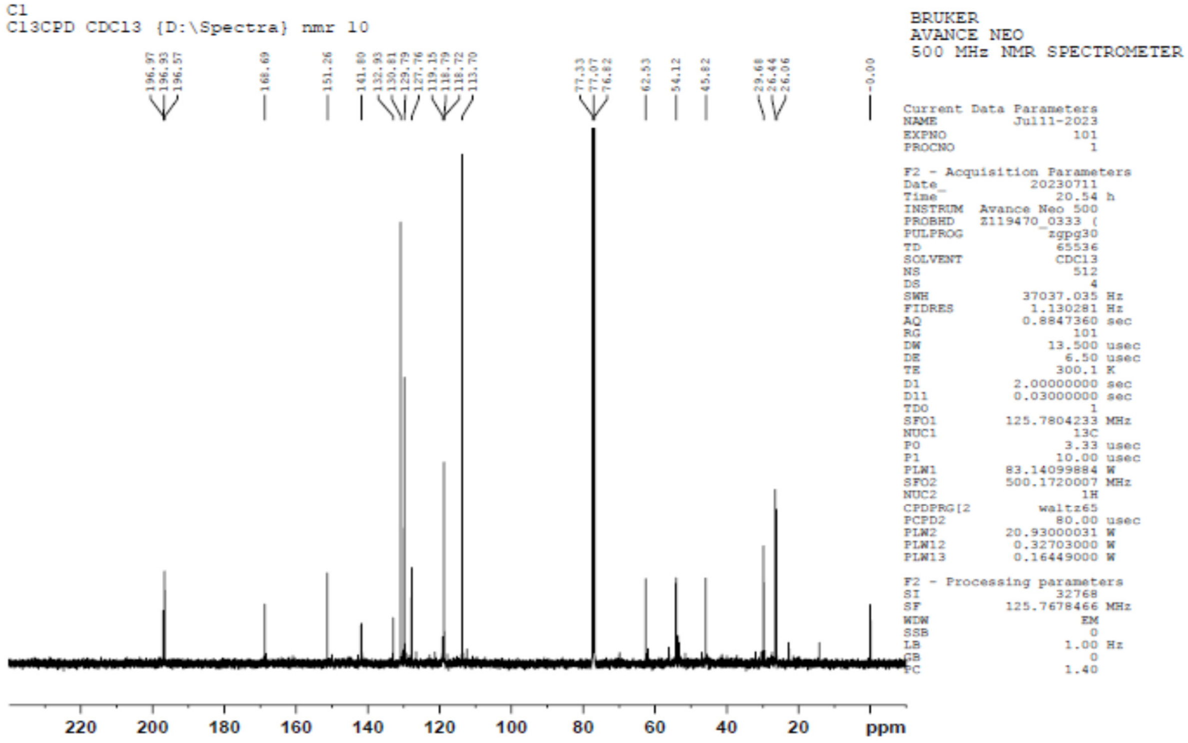
C1
C13CPD CDCl3 {D:\Spectra} nmr 10
196.97
196.93
196.57
168.69
151.26
141.80
132.93
130.81
129.79
127.76
119.15
118.79
118.72
113.70
77.33
77.07
76.82
62.53
54.12
45.82
29.68
26.44
26.06
-0.00
BRUKER
AVANCE NEO
500 MHz NMR SPECTROMETER
Current Data Parameters
NAME Jul11-2023
EXPNO 101
PROCNO 1
F2 - Acquisition Parameters
Date_ 20230711
Time 20.54 h
INSTRUM Avance Neo 500
PROBHD Z119470_0333 (
PULPROG zgpg30
TD 65536
SOLVENT CDCl3
NS 512
DS 4
SWH 37037.035 Hz
FIDRES 1.130281 Hz
AQ 0.8847360 sec
RG 101
DW 13.500 usec
DE 6.50 usec
TE 300.1 K
D1 2.00000000 sec
D11 0.03000000 sec
TD0 1
SFO1 125.7804233 MHz
NUC1 13C
P0 3.33 usec
P1 10.00 usec
PLW1 83.14099884 W
SFO2 500.1720007 MHz
NUC2 1H
CPDPRG[2 waltz65
PCPD2 80.00 usec
PLW2 20.93000031 W
PLW12 0.32703000 W
PLW13 0.16449000 W
F2 - Processing parameters
SI 32768
SF 125.7678466 MHz
WDW EM
SSB 0
LB 1.00 Hz
GB 0
PC 1.40
220
200
180
160
140
120
100
80
60
40
20
ppm

C-2

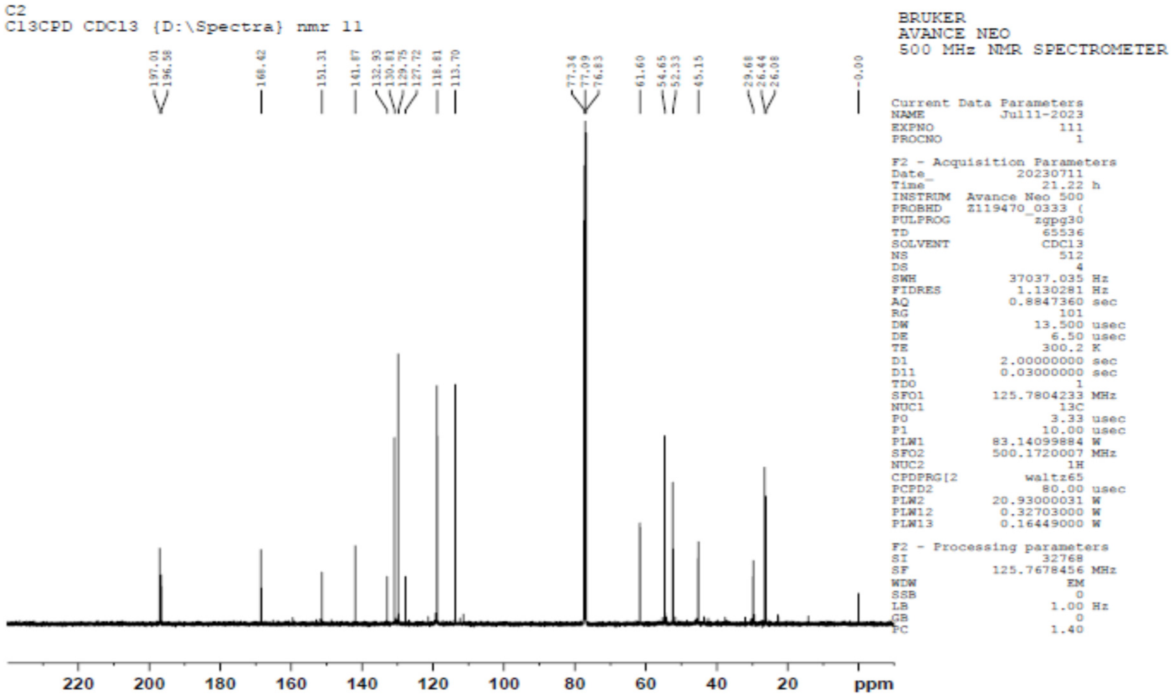
C2
C13CPD CDCl3 {D:\Spectra} nmr 11
197.01
196.58
168.42
151.31
141.87
132.93
130.81
129.75
127.72
118.81
113.70
77.34
77.09
76.83
61.60
54.65
52.33
45.15
29.68
26.44
26.08
-0.00
BRUKER
AVANCE NEO
500 MHz NMR SPECTROMETER
Current Data Parameters
NAME Jul11-2023
EXPNO 111
PROCNO 1
F2 - Acquisition Parameters
Date_ 20230711
Time 21.22 h
INSTRUM Avance Neo 500
PROBHD Z119470_0333 (
PULPROG zgpg30
TD 65536
SOLVENT CDCl3
NS 512
DS 4
SWH 37037.035 Hz
FIDRES 1.130281 Hz
AQ 0.8847360 sec
RG 101
DW 13.500 usec
DE 6.50 usec
TE 300.2 K
D1 2.00000000 sec
D11 0.03000000 sec
TD0 1
SFO1 125.7804233 MHz
NUC1 13C
P0 3.33 usec
P1 10.00 usec
PLW1 83.14099884 W
SFO2 500.1720007 MHz
NUC2 1H
CPDPRG[2 waltz65
PCPD2 80.00 usec
PLW2 20.93000031 W
PLW12 0.32703000 W
PLW13 0.16449000 W
F2 - Processing parameters
SI 32768
SF 125.7678456 MHz
WDW EM
SSB 0
LB 1.00 Hz
GB 0
PC 1.40
220
200
180
160
140
120
100
80
60
40
20
ppm

C-3

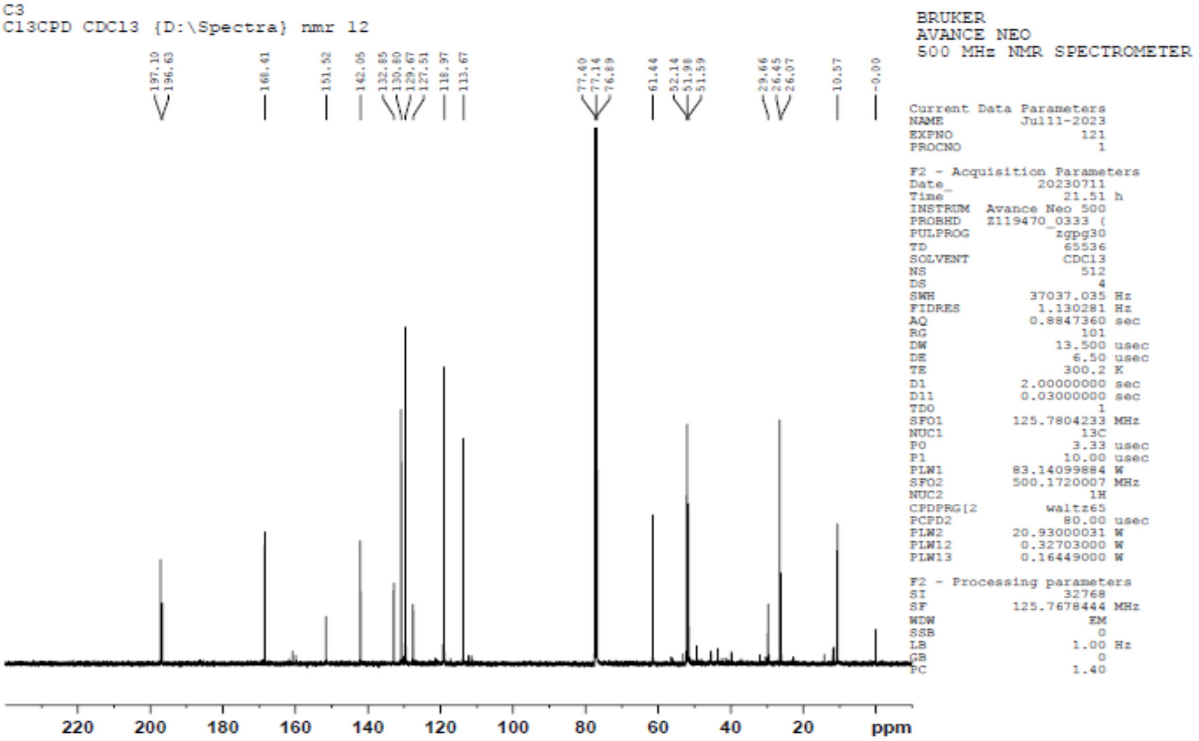
C3
C13CPD CDCl3 {D:\Spectra} nmr 12
BRUKER
AVANCE NEO
500 MHz NMR SPECTROMETER
197.10
196.63
168.41
151.52
142.05
132.85
130.80
129.67
127.51
118.97
113.67
77.40
77.14
76.89
61.44
52.14
51.98
51.59
29.66
26.45
26.07
10.57
-0.00
Current Data Parameters
NAME Jul11-2023
EXPNO 121
PROCNO 1
F2 - Acquisition Parameters
Date_ 20230711
Time 21.51 h
INSTRUM Avance Neo 500
PROBHD Z119470_0333 (
PULPROG zgpg30
TD 65536
SOLVENT CDCl3
NS 512
DS 4
SWH 37037.035 Hz
FIDRES 1.130281 Hz
AQ 0.8847360 sec
RG 101
DW 13.500 usec
DE 6.50 usec
TE 300.2 K
D1 2.00000000 sec
D11 0.03000000 sec
TD0 1
SFO1 125.7804233 MHz
NUC1 13C
P0 3.33 usec
P1 10.00 usec
PLW1 83.14099884 W
SFO2 500.1720007 MHz
NUC2 1H
CPDPRG[2 waltz65
PCPD2 80.00 usec
PLW2 20.93000031 W
PLW12 0.32703000 W
PLW13 0.16449000 W
F2 - Processing parameters
SI 32768
SF 125.7678444 MHz
WDW EM
SSB 0
LB 1.00 Hz
GB 0
PC 1.40
220
200
180
160
140
120
100
80
60
40
20
ppm

C-4

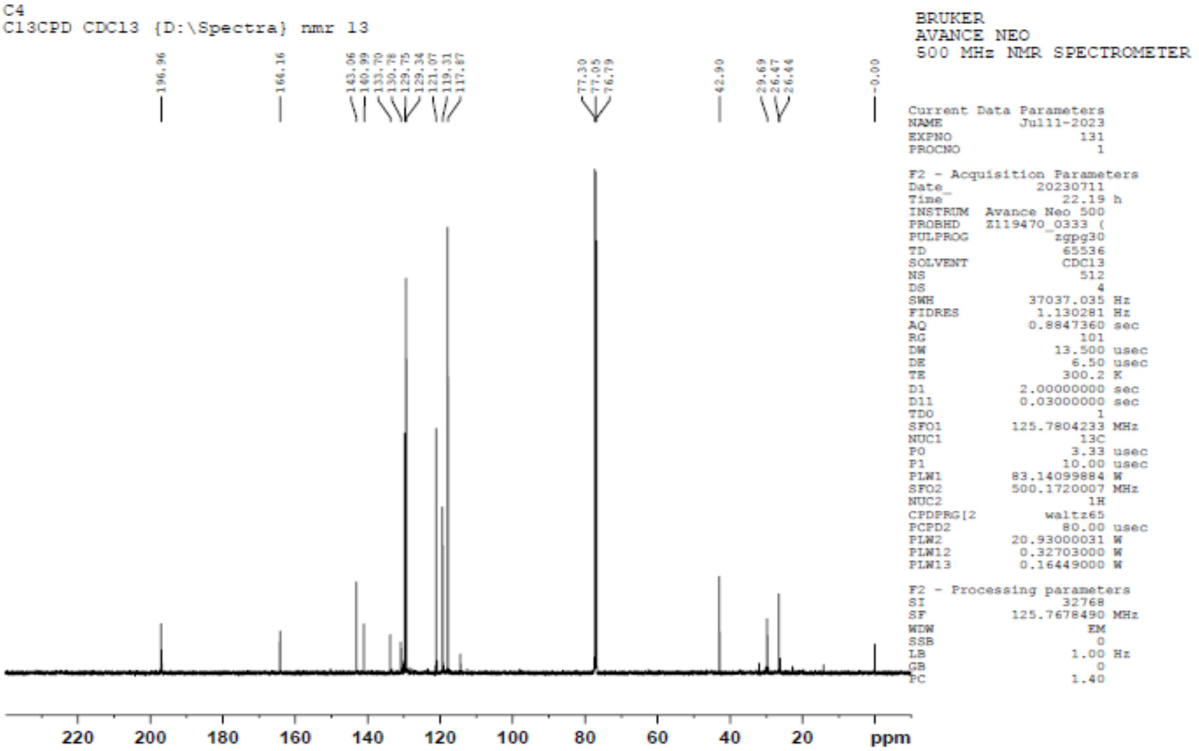
C4
C13CPD CDCl3 {D:\Spectra} nmr 13
BRUKER
AVANCE NEO
500 MHz NMR SPECTROMETER
196.96
164.16
143.06
140.99
133.70
130.78
129.75
129.34
121.07
119.31
117.87
77.30
77.05
76.79
42.90
29.69
26.47
26.44
-0.00
Current Data Parameters
NAME Jul11-2023
EXPNO 131
PROCNO 1
F2 - Acquisition Parameters
Date_ 20230711
Time 22.19 h
INSTRUM Avance Neo 500
PROBHD Z119470_0333 (
PULPROG zgpg30
TD 65536
SOLVENT CDCl3
NS 512
DS 4
SWH 37037.035 Hz
FIDRES 1.130281 Hz
AQ 0.8847360 sec
RG 101
DW 13.500 usec
DE 6.50 usec
TE 300.2 K
D1 2.00000000 sec
D11 0.03000000 sec
TD0 1
SFO1 125.7804233 MHz
NUC1 13C
P0 3.33 usec
P1 10.00 usec
PLW1 83.14099884 W
SFO2 500.1720007 MHz
NUC2 1H
CPDPRG[2 waltz65
PCPD2 80.00 usec
PLW2 20.93000031 W
PLW12 0.32703000 W
PLW13 0.16449000 W
F2 - Processing parameters
SI 32768
SF 125.7678490 MHz
WDW EM
SSB 0
LB 1.00 Hz
GB 0
PC 1.40
220
200
180
160
140
120
100
80
60
40
20
ppm

C-5

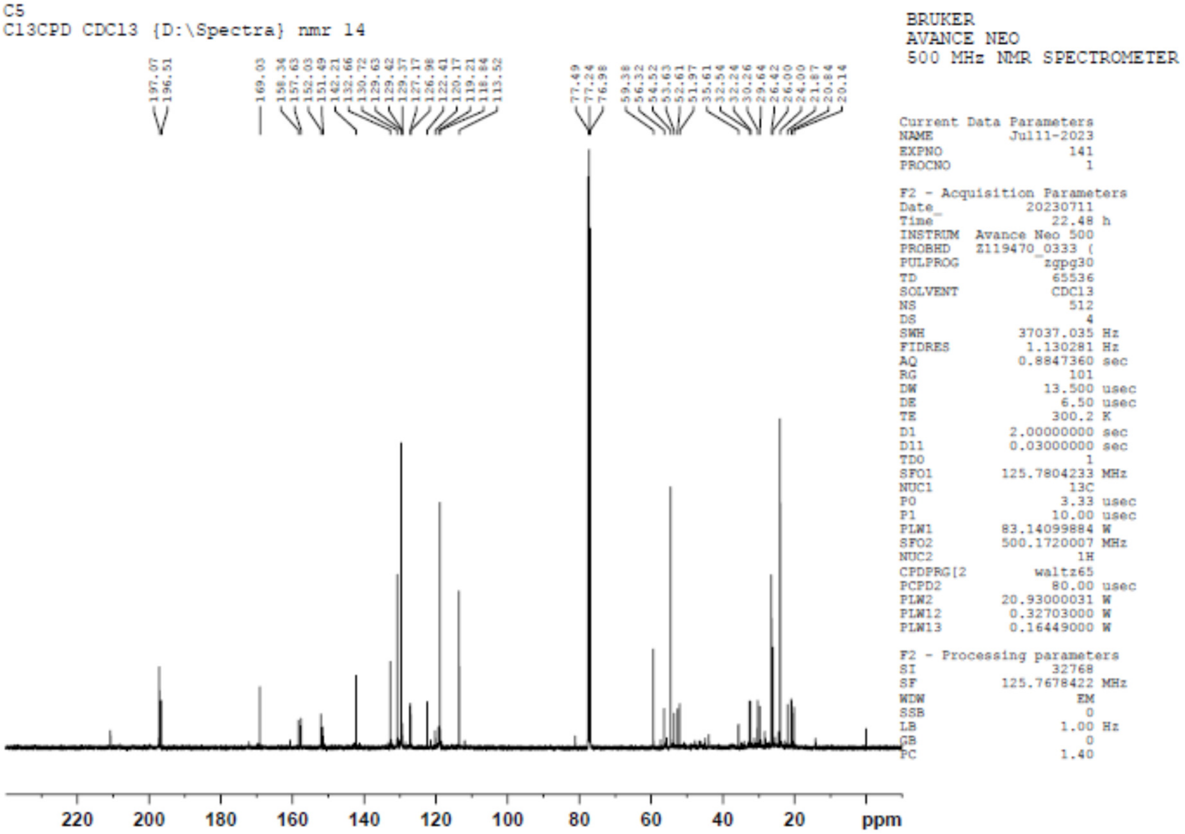
C5
C13CPD CDCl3 {D:\Spectra} nmr 14
BRUKER
AVANCE NEO
500 MHz NMR SPECTROMETER
197.07
196.51
169.03
158.34
157.63
152.03
151.49
142.21
132.66
130.72
129.63
129.42
129.37
127.17
126.98
122.41
120.17
119.21
118.84
113.52
77.49
77.24
76.98
59.38
56.32
54.52
53.63
52.61
51.97
35.61
32.54
32.24
30.26
29.64
26.42
26.00
24.00
21.87
20.84
20.14
Current Data Parameters
NAME Jul11-2023
EXPNO 141
PROCNO 1
F2 - Acquisition Parameters
Date_ 20230711
Time_ 22.48 h
INSTRUM Avance Neo 500
PROBHD Z119470_0333 (
PULPROG zgpg30
TD 65536
SOLVENT CDCl3
NS 512
DS 4
SWH 37037.035 Hz
FIDRES 1.130281 Hz
AQ 0.8847360 sec
RG 101
DW 13.500 usec
DE 6.50 usec
TE 300.2 K
D1 2.00000000 sec
D11 0.03000000 sec
TD0 1
SFO1 125.7804233 MHz
NUC1 13C
P0 3.33 usec
P1 10.00 usec
PLW1 83.14099884 W
SFO2 500.1720007 MHz
NUC2 1H
CPDPRG[2 waltz65
PCPD2 80.00 usec
PLW2 20.93000031 W
PLW12 0.32703000 W
PLW13 0.16449000 W
F2 - Processing parameters
SI 32768
SF 125.7678422 MHz
WDW EM
SSB 0
LB 1.00 Hz
GB 0
PC 1.40
220
200
180
160
140
120
100
80
60
40
20
ppm
