# Supplementary material for: Synthesis, Characterization, and Docking Studies of Some New Chalcone Derivatives to Alleviate Skin Damage Due to UV Light
Source: Molecules. 2025 Feb 25;30(5):1057. doi: 10.3390/molecules30051057 (PMC11901719; doi:10.3390/molecules30051057)
Supplement: Supplementary file 1 [file molecules-30-01057-s001.zip › Supplementary Materials Figures.pdf]

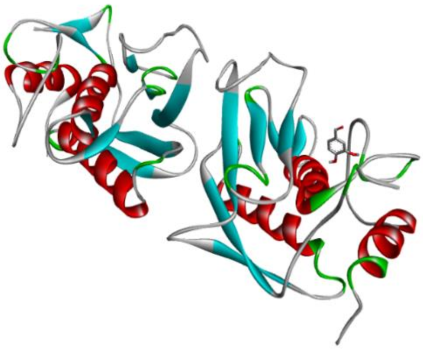

A

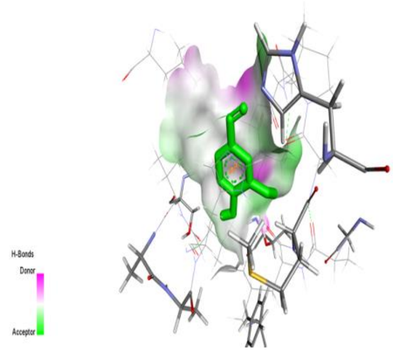

B

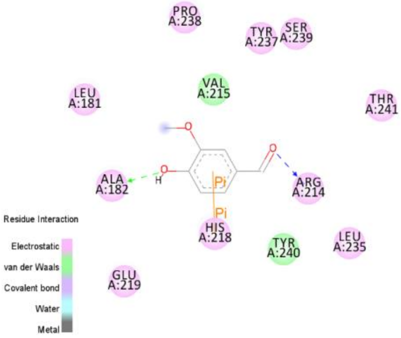

C

Figure S1. Molecular interaction of Vanillin with PDB: 3shi of MMP1. (A) & (B) are 3D view, while (C) is showing 2D view of molecular interaction

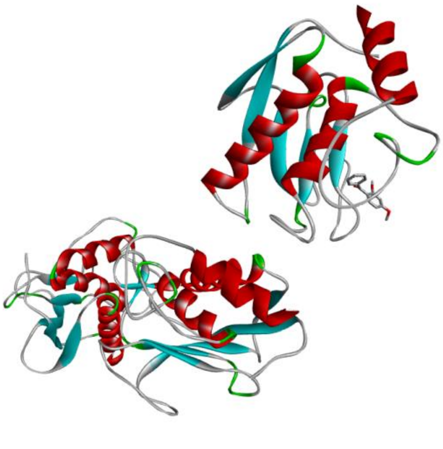

A

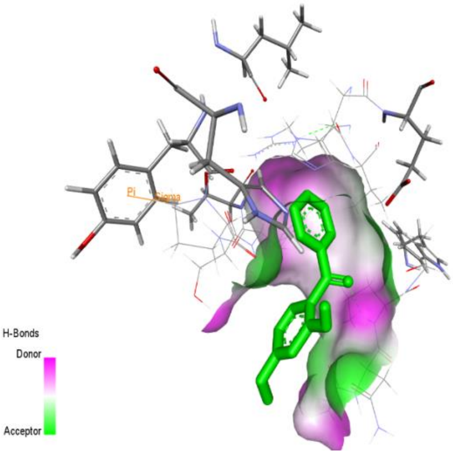

B

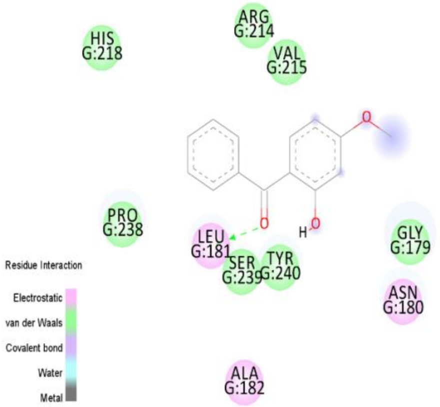

C

Figure S2. Molecular interaction of Oxybenzone with PDB: 3shi of MMP1. (A) & (B) are 3D view, while (C) is showing 2D view of molecular interaction

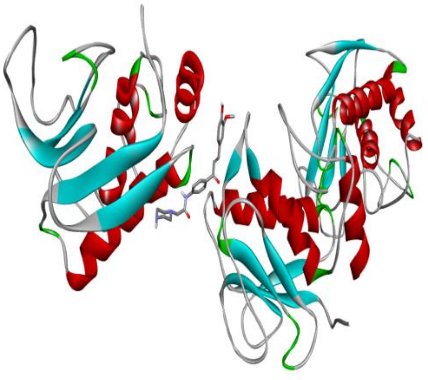

A

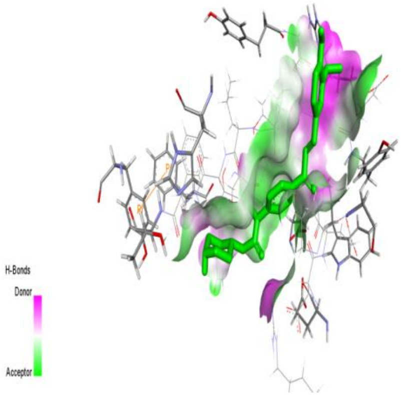

B

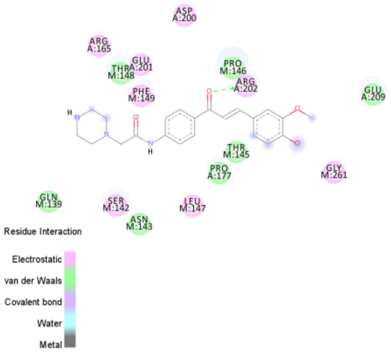

C

Figure S3. Molecular interaction of C1 with PDB: 3shi of MMP1. (A) & (B) are 3D view, while (C) is showing 2D view of molecular interaction

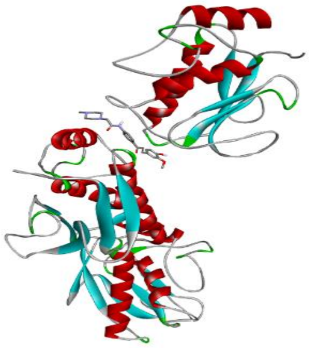

A

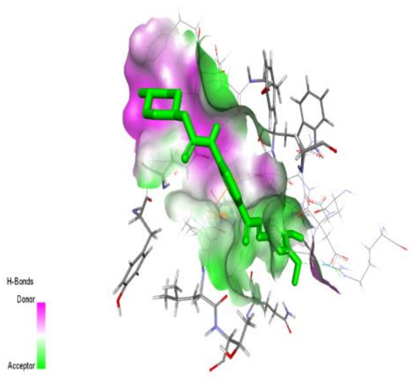

B

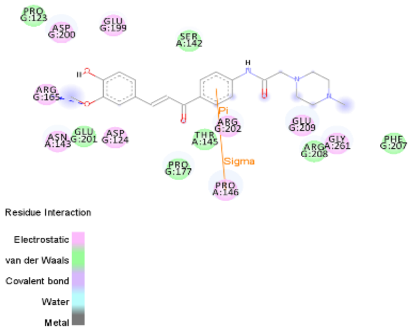

C

Figure S4. Molecular interaction of C2 with PDB: 3shi of MMP1. (A) & (B) are 3D view, while (C) is showing 2D view of molecular interaction

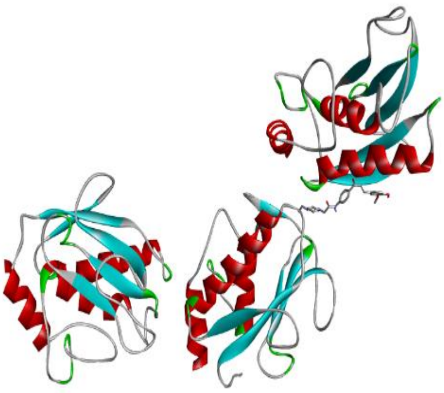

A

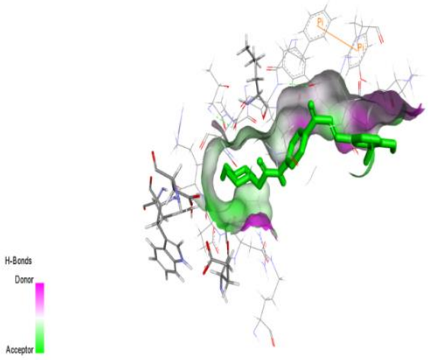

B

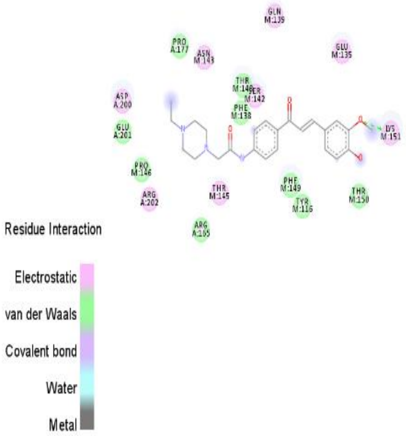

C

FigureS 5. Molecular interaction of C3 with PDB: 3shi of MMP1. (A) & (B) are 3D view, while (C) is showing 2D view of molecular interaction

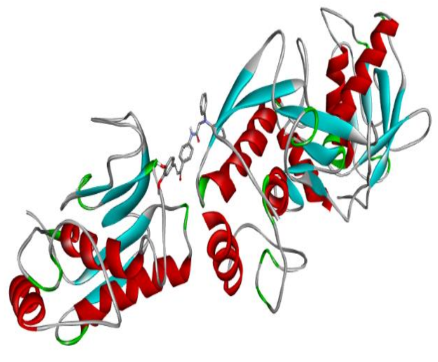

A

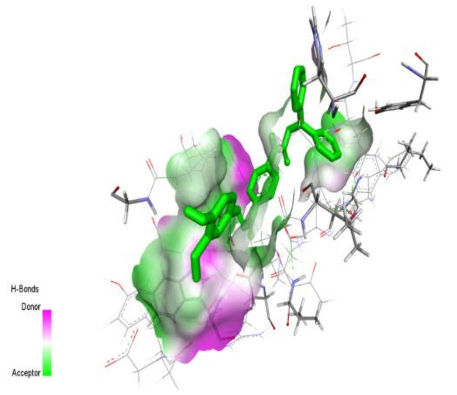

B

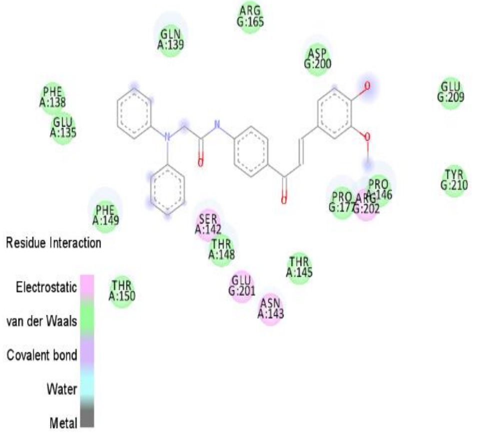

C

Figure S6. Molecular interaction of **C4** with PDB: 3shi of MMP1. (A) & (B) are 3D view, while (C) is showing 2D view of molecular interaction

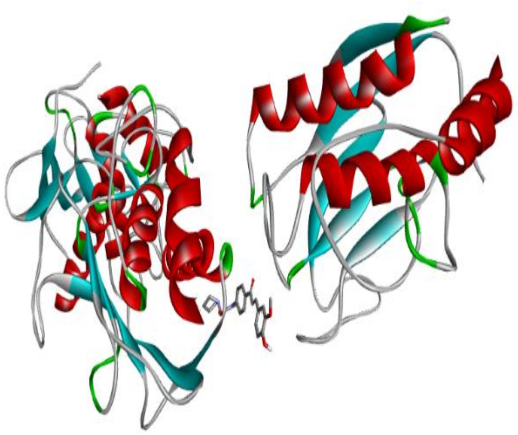

A

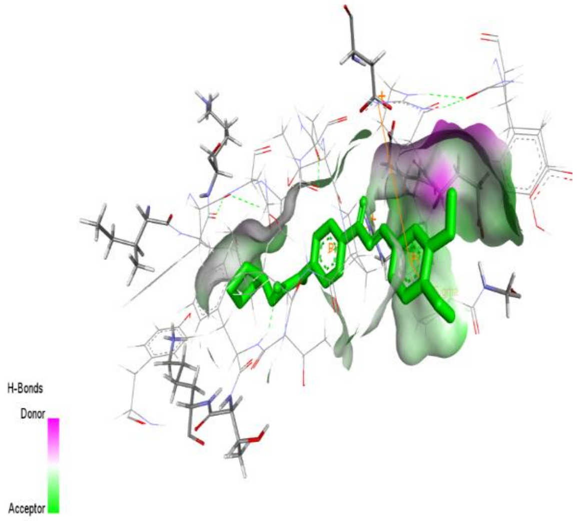

B

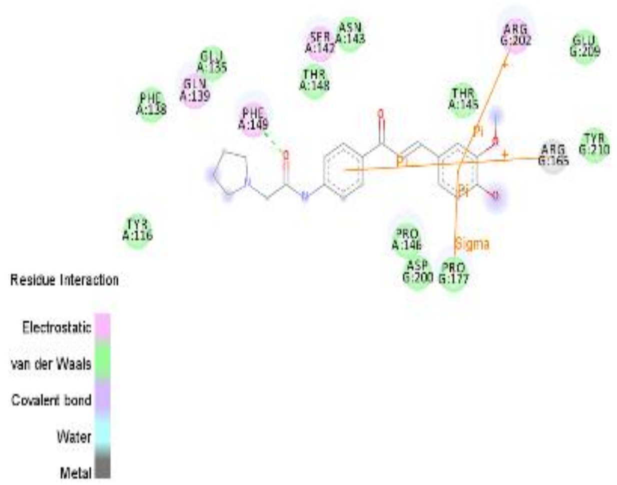

C

Figure S7. Molecular interaction of **C5** with PDB: 3shi of MMP1. (A) & (B) are 3D view, while (C) is showing 2D view of molecular interaction

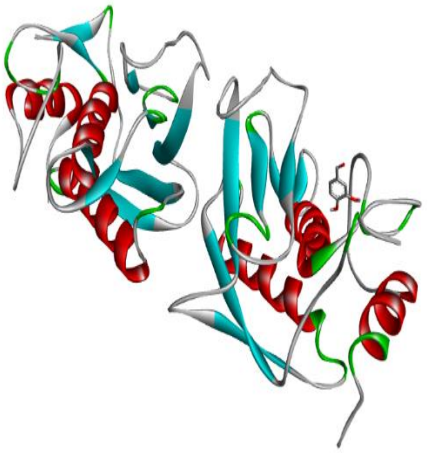

A

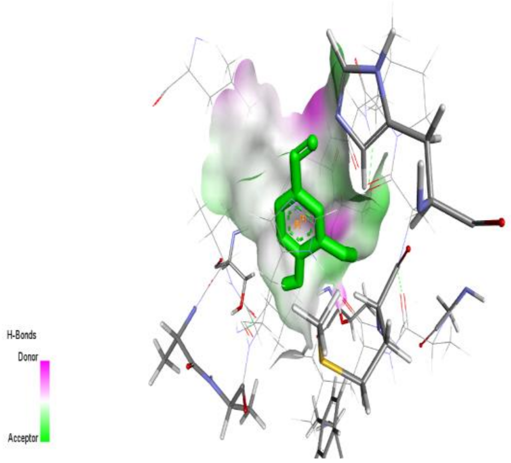

B

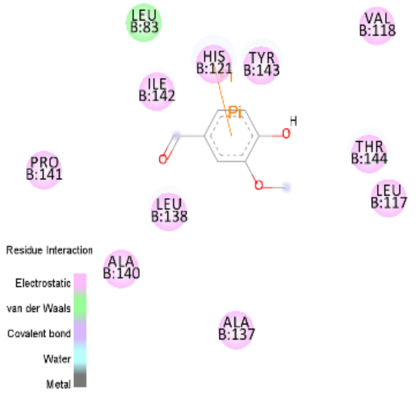

C

Figure S8. Molecular interaction of **Vanilin** with PDB: 8H78 of MMP2. (A) & (B) are 3D view, while (C) is showing 2D view of molecular interaction

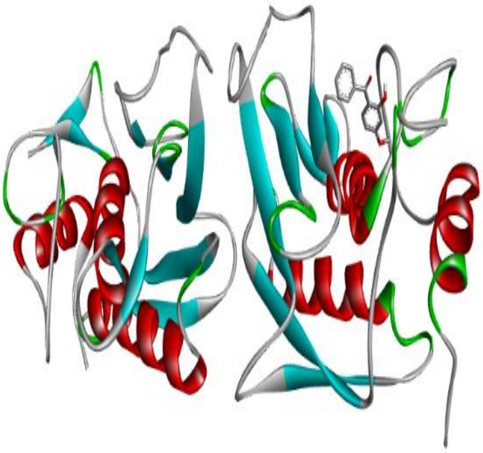

A

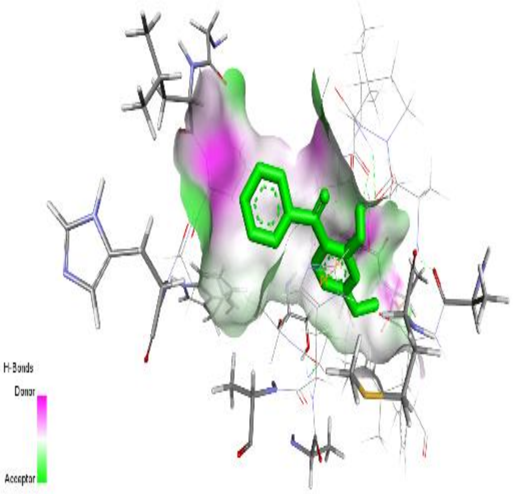

B

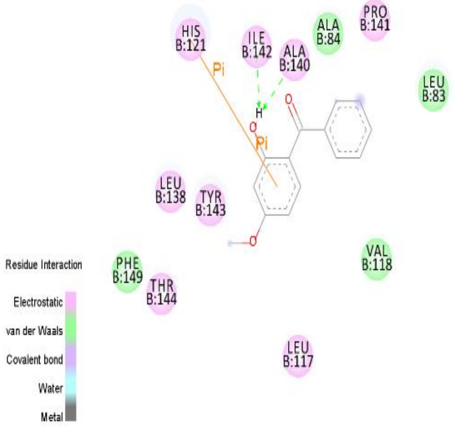

C

Figure S9. Molecular interaction of **Oxybenzone** with PDB: 8H78 of MMP2. (A) & (B) are 3D view, while (C) is showing 2D view of molecular interaction

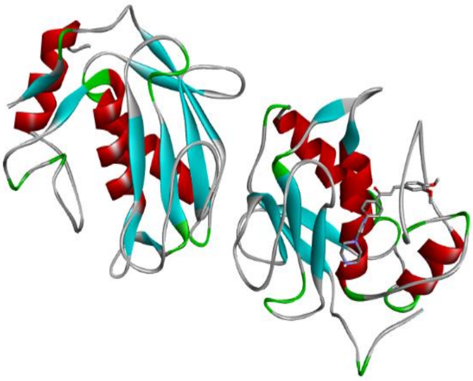

A

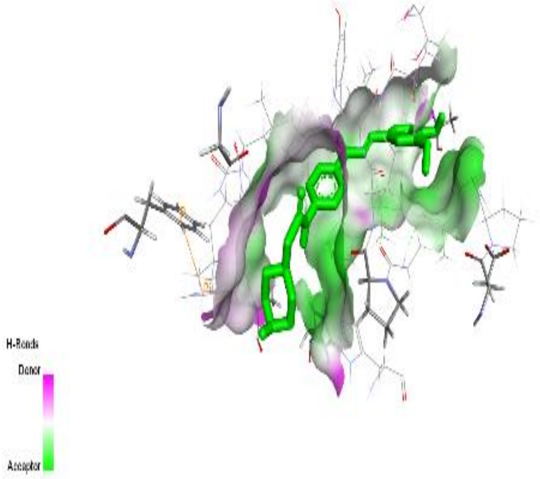

B

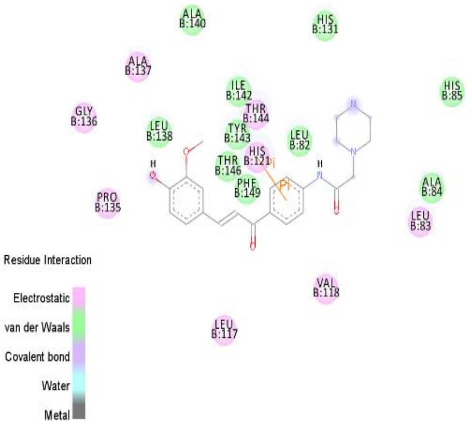

C

Figure S10. Molecular interaction of **C1** with PDB: 8H78 of MMP2. (A) & (B) are 3D view, while (C) is showing 2D view of molecular interaction

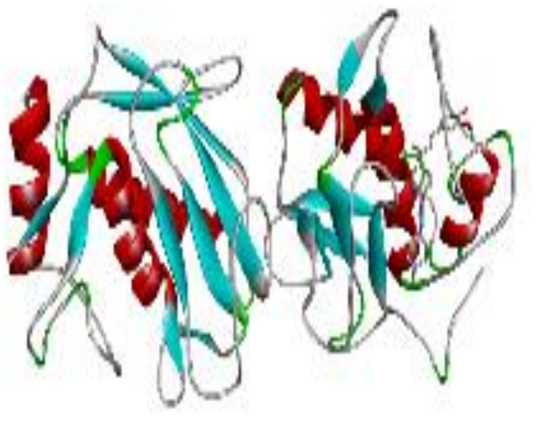 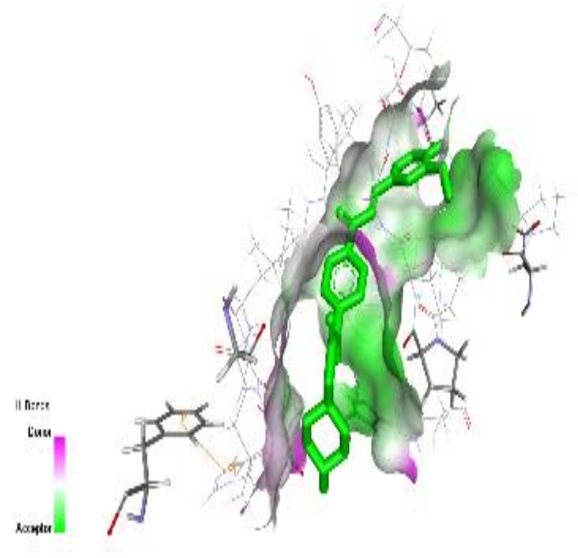

A **B**

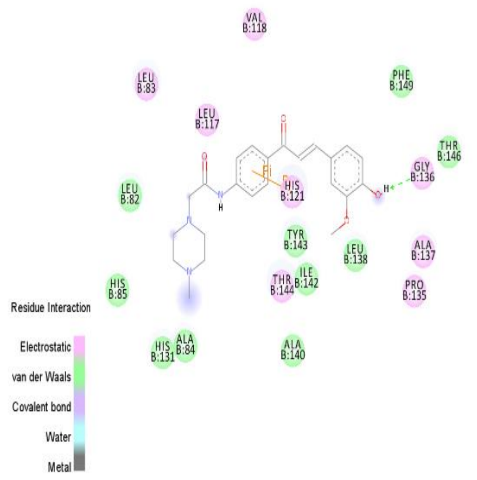

C

Figure S11. Molecular interaction of **C2** with PDB: 8H78 of MMP2. (A) & (B) are 3D view, while (C) is showing 2D view of molecular interaction

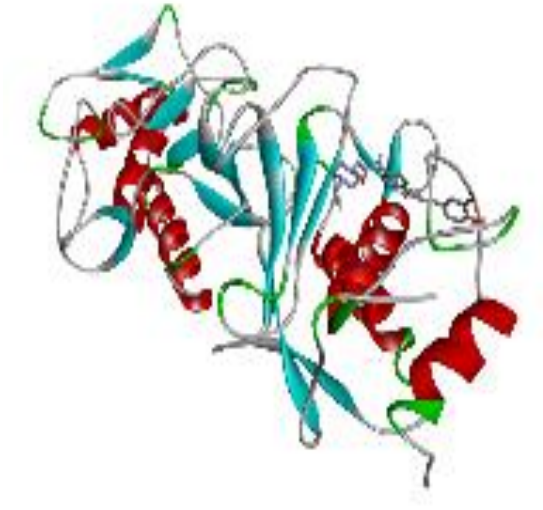

A

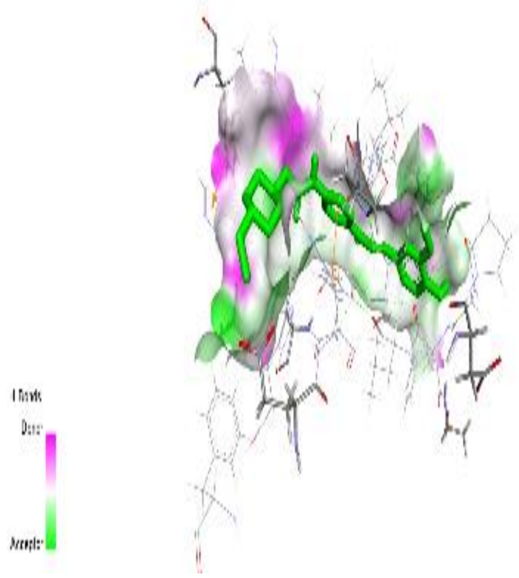

B

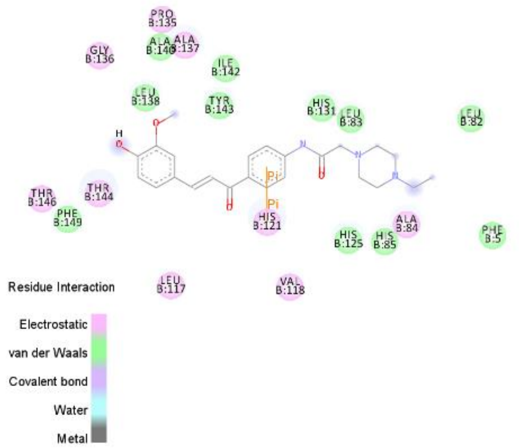

C

Figure S12. Molecular interaction of **C3** with PDB: 8H78 of MMP2. (A) & (B) are 3D view, while (C) is showing 2D view of molecular interaction

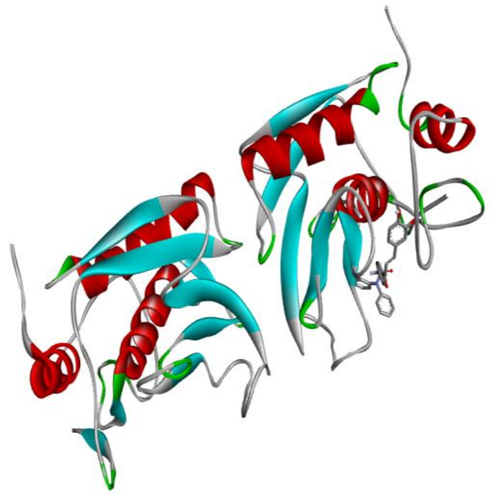

A

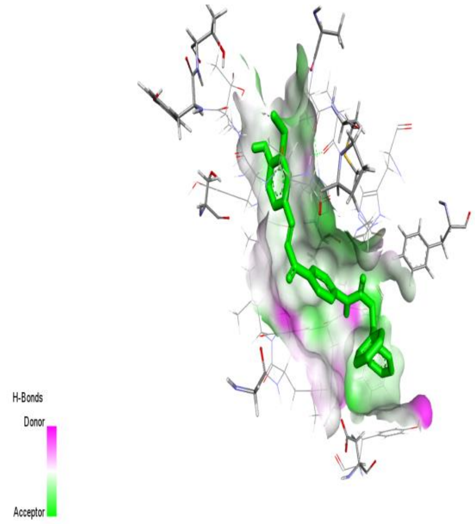

B

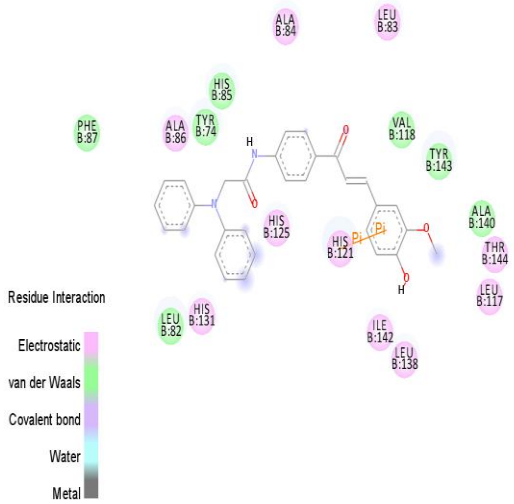

C

Figure S13. Molecular interaction of **C4** with PDB: 8H78 of MMP2. (A) & (B) are 3D view, while (C) is showing 2D view of molecular interaction

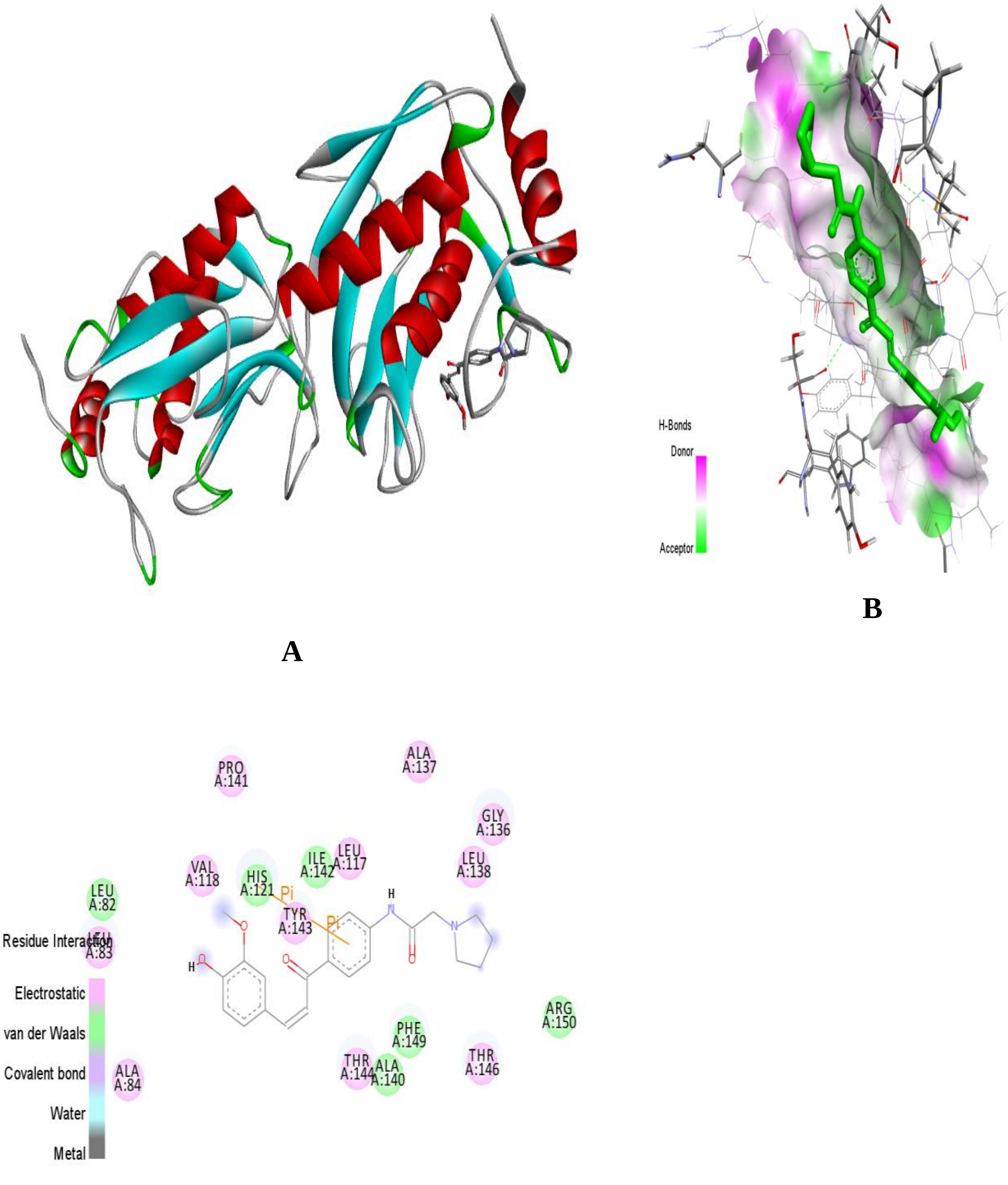

A

B

C

Figure S14. Molecular interaction of **C5** with PDB: 8H78 of MMP2. (A) & (B) are 3D view, while (C) is showing 2D view of molecular interaction
