# Supplementary material for: Synthesis, Characterization, and Docking Studies of Some New Chalcone Derivatives to Alleviate Skin Damage Due to UV Light
Source: Molecules. 2025 Feb 25;30(5):1057. doi: 10.3390/molecules30051057 (PMC11901719; doi:10.3390/molecules30051057)
Supplement: Supplementary file 1 [file molecules-30-01057-s001.zip › Supplementary Materials Tables.pdf]

Table S1: Physical Parameters and Pictorial Presentation of Nanoemulsions

**Parameters**	**Observations**	**Pictorial Presentation of Prepared Nanoemulsion**
Color	Cream	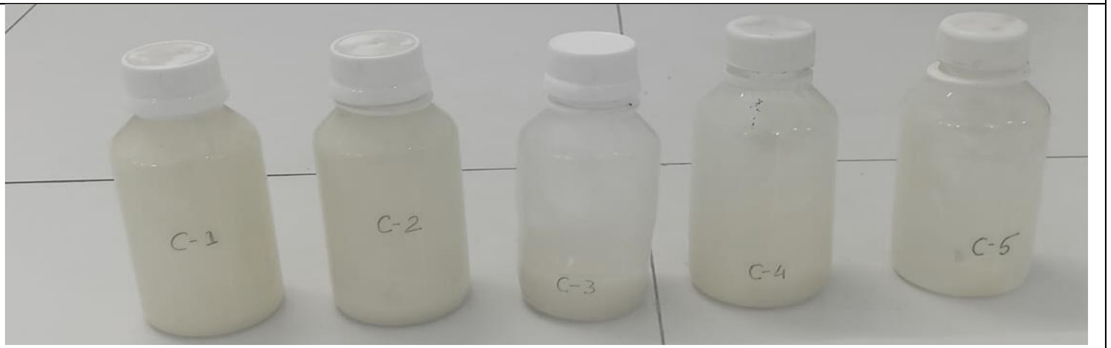
Odour	Pleasant	

TableS2: Physicochemical characteristics of Nanoemulsions

**S.No.**	**Parameters analyzed**	**C-1NE**	**C-2NE**	**C-3NE**	**C-4NE**	**C-5NE**	**Blank**
1	Particle Size, nm	172.30	183.00	173.78	180.77	180.81	172.57
2	Polydispersity index	0.14	0.12	0.13	0.11	0.15	0.12
3	Zeta Potential, mV	-39.30	-38.43	-37.90	-38.18	-37.23	-35.87
4	pH	5.11	5.16	5.10	5.09	5.12	5.00
5	Viscosity, cP	2.00	2.01	2.09	2.07	2.08	2.12

Table S3. Score for skin reaction

Group Reaction	Score of Skin reaction											
	Base		C-1NE		C-2NE		C-3NE		C-4NE		C-5NE	
	24 h	72h	24 h	72h	24 h	72h	24 h	72h	24 h	72h	24 h	72h
Erythema	1	0	1	1	2	1	1	1	1	1	1	1
Edema	1	0	1	0	0	2	1	0	1	1	1	1
Erythema	1	1	1	0	1	1	2	1	1	0	1	1
Edema	1	0	2	0	0	1	0	0	0	0	0	0
Erythema	2	1	2	1	1	1	1	1	1	0	1	1
Edema	1	0	1	0	1	0	0	0	0	0	1	0
Erythema	1	0	2	1	1	1	2	1	2	0	1	1
Edema	1	1	1	0	0	1	1	0	0	1	0	1
Erythema	1	1	2	0	0	1	1	1	1	1	1	1
Edema	1	1	1	0	2	1	0	0	0	0	2	1
**PII**	**16/20=0.80**		**17/20=0.85**		**18/20=0.90**		**14/20=0.70**		**11/20=0.55**		**17/20=0.85**	

For Erythema and edema 0 = No erythema and edema, 1 = very slightlyerythema and edema, 2 = Well definederythema and edema

Table S4.*In vivo* SPF values

S. No.	Animal's Groups	SPF values
1	Group 1	0.23
2	Group 2	9.78
3	Group 3	2.59
4	Group 4	2.73
5	Group 5	5.39
6	Group 6	7.20
7	Group 7	3.71

Table S5. Erythema Scores with diameter range

Erythema Score	Description	Diameter Range
0	No Erythema	≤ 20.00 mm
1	Very Little Erythema	20.01 - 25.00 mm
2	Clearly Defined Erythema	25.01 - 30.00 mm
3	Moderate to Severe Erythema	30.01 - 35.00 mm
4	Shaping Crust	diameter ≥35
