# Supplementary figures and images for: Synthesis, Characterization, and Docking Studies of Some New Chalcone Derivatives to Alleviate Skin Damage Due to UV Light
Source: Molecules. 2025 Feb 25;30(5):1057. doi: 10.3390/molecules30051057 (PMC11901719; doi:10.3390/molecules30051057)

SAIF, PANJAB UNIVERSITY, CHANDIGARH

SYNAPT-XS#DBA064

11-Jul-2023

C\_4 11 (0.181) Cm (4.25)

1: TOF MS ES+  
6.63e4

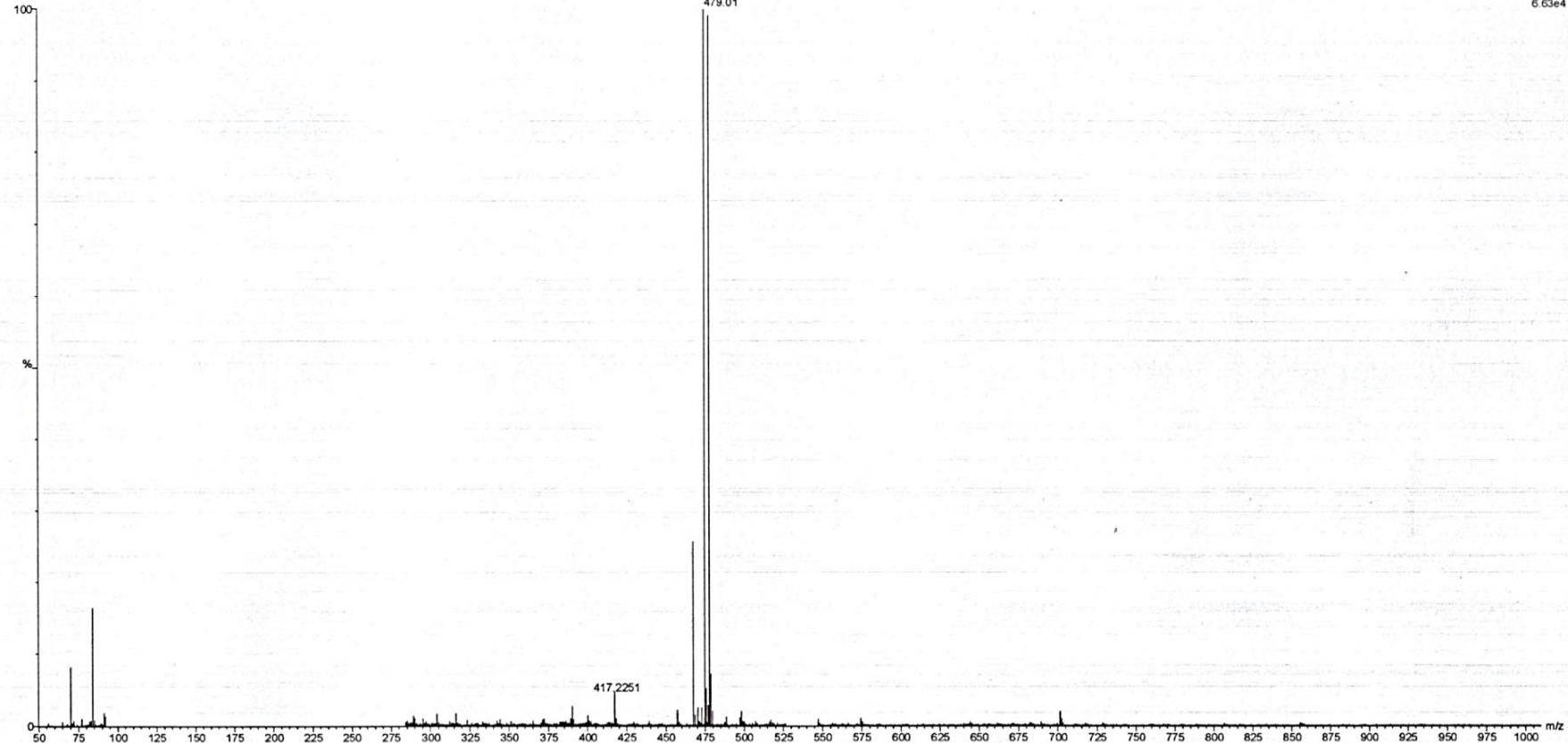

Supplement: Supplementary file 1 [file molecules-30-01057-s001.zip › Mass spectra of C4-Most potent compound.pdf]
